# Element-Free Galerkin Method for Analyzing Size-Dependent Thermally Induced Free Vibration Characteristics of Functionally Graded Magneto-Electro-Elastic Doubly Curved Microscale Shells

**DOI:** 10.3390/ma19081494

**Published:** 2026-04-08

**Authors:** Chih-Ping Wu, Meng-Jung Liu

**Affiliations:** Department of Civil Engineering, National Cheng Kung University, Tainan City 70101, Taiwan

**Keywords:** consistent couple stress theory, differential reproducing kernel interpolants, doubly curved microscale shells, element-free Galerkin method, functionally graded material, thermally induced free vibration

## Abstract

Within the framework of consistent couple stress theory (CCST) and employing Hamilton’s principle, we derive a Galerkin weak formulation to analyze the three-dimensional (3D) size-dependent free vibration characteristics of a simply supported, functionally graded (FG) magneto-electro-elastic (MEE) doubly curved (DC) microscale shell subjected to a uniform temperature change. Incorporating the differential reproducing kernel (DRK) interpolants into the weak formulation, we further develop an element-free Galerkin (EFG) method. The microscale shell of interest is composed of two-phase MEE materials, and its material properties are assumed to vary through its thickness according to a power-law distribution of the volume fractions of the constituents. The results show that the natural frequency solutions obtained using the EFG method are in excellent agreement with the reported 3D solutions for laminated composite and FG-MEE macroscale plates, with the material length-scale parameter and the inverse of the curvature radii set to zero. The effects of the material length-scale parameter, temperature change, inhomogeneity index, and mid-surface radius and length-to-thickness ratios on the FG-MEE microscale shell’s free vibration characteristics in a thermal environment are examined and appear to be significant.

## 1. Introduction

Magneto-electro-elastic (MEE) materials [1], due to their coupling among mechanical, electric, and magnetic fields, can convert energy among these fields, enabling a wide range of applications in advanced industries. These include sensors that detect changes in mechanical, electrical, and magnetic fields [2]; energy-harvesting devices that convert ambient vibrations into electrical and magnetic energy [3,4]; and actuators that exploit coupling among multiple fields for precise control [5]. The analysis of the coupling of magneto-electro-mechanical behaviors in laminated composite (LC) and functionally graded (FG) MEE beam, plate, and shell-like structures has thus attracted considerable attention.

Several articles have reported results on the magneto-electro-mechanical coupling behavior of LC and FG macroscale MEE structures. Using the first-order shear deformation theory (FSDT), Zhang et al. [6] presented results for the mechanical behavior of FG-MEE plates and shells using a finite element method (FEM). Accounting for porosity and thermal effects, Zhao et al. [7] used FSDT to analyze the mechanical response of FG-MEE porous cylindrical shells in a thermal environment. Based on the refined shear deformation theory (RSDT), Moita et al. [8] developed a higher-order FEM model to investigate the free vibration and static bending behaviors of LC-MEE plates. Tornabene et al. [9] derived an equivalent layer-wise formulation to study the magneto-electro-mechanical coupling behavior of LC-MEE doubly curved (DC) shells. Within the framework of three-dimensional (3D) classical elasticity theory, Bhangale and Ganesan [10] and Wu et al. [11] developed semi-analytical FEM and meshless methods, respectively, to analyze the mechanical behavior of simply supported FG-MEE plates and shells. Pan and Heyliger [12], Brischetto and Cesare [13], Brischetto et al. [14], Wu et al. [15], Wu and Tsai [16], and Tsai and Wu [17] presented 3D exact solutions for various mechanical behaviors of LC- and FG-MEE plates and shells using classical analytical approaches, including the state space, modified Pagano, and perturbation methods. Vinyas [18] conducted a comprehensive survey of computational models to analyze the mechanical behavior of smart LC- and FG-MEE macroscale structures.

MEE materials also enable the miniaturization of components and structures in high-tech fields, including filtering antennas and inductors [19], micro-electro-mechanical systems (MEMSs) [20], vibration isolators [21], soft robotics [22], and soft sensors and actuators [23]. It is well known that the mechanical behavior of microscale devices and structures differs from that of macroscale devices and structures due to size-dependent effects. To account for these effects in classical continuum mechanics (CCM) models, several non-CCM theories have been reported, including the couple stress theory and its consistent and modified versions [24,25,26], the strain gradient theory and its consistent and modified versions [27,28,29,30], Eringen’s nonlocal elasticity theory [31], the micropolar theory [32], and the doublet mechanics theory [33]. Finally, several comprehensive surveys on the development of various non-CCM theories and their applications have been presented [34,35,36,37,38,39,40]. Among these non-CCM theories, the modified and consistent couple stress theories (MCSTs and CCSTs) are more widely used than the others because they require only one material-length scale parameter for calibration. The relevant two-dimensional (2D) analyses based on the MCST and CCST are surveyed as follows:

Mehralian and Beni [41] investigated the thermo-electro-mechanical buckling behavior of cylindrical nanoshells resting on an elastic foundation by incorporating the kinematics model of Love’s classical shell theory into the MCST. Based on the MCST, Lou et al. [42] reformulated the classical FSDT to account for size-dependent effects and used it to analyze the post-buckling response of piezoelectric hybrid microplates subjected to thermo-electro-mechanical loads. In conjunction with the MCST and the kinematics model of the RSDT, Abazid and Sobhy [43] examined the static bending response of an FG piezoelectric microplate resting on an elastic foundation. Combining the kinematics model of the sinusoidal shear deformation theory (SSDT) and the MCST, Zhang et al. [44] analyzed the static buckling behavior of a sandwich microplate. Accounting for the thickness stretching effect in the MCST-based SSDT, Dehsaraji et al. [45] presented results on the thermo-electro-mechanical buckling analysis of FG piezoelectric micro/nano-shells. Finally, based on the CCST, Wu and Hsu [46] developed a 2D unified size-dependent theory to analyze the free vibration characteristics of a simply supported FG microplate subjected to magneto-electro-thermo-mechanical loads. Wu and Hsu [26] have proved that the difference between the weak formulation of the MCST and that of the CCST is minor, and the results obtained from these two theories closely agree with each other.

On one hand, because the differential operator applied to the total stress tensor in the stress equilibrium equations for the CCST aligns with that used for the symmetric force-stress tensor in classical elasticity theory, the CCST is more practical to use than the MCST. On the other hand, the highest-order derivatives of the field variables in the current study are third order. The differential reproducing kernel (DRK), developed by the first author’s research group [47], consists of a higher-order polynomial function set that naturally exhibits a greater capacity for smoothly simulating the variations in field variables than the lower-order Lagrange functions used to construct the shape functions in traditional FEMs. Therefore, the element-free Galerkin (EFG) method with DRK interpolants becomes an efficient numerical approach for analyzing the current thermally induced physical problems.

Following the review above, we found that 3D solutions for the coupling behavior of LC- and FG-MEE microscale shells in thermal environments are limited in the literature. To provide a reference for evaluating 2D numerical solutions and understanding how key factors influence the free-vibration characteristics of LC- and FG-MEE DC microscale shells in thermal environments, such as the material length-scale parameter, temperature change, inhomogeneity index, mid-surface radius ratio, and length-to-thickness ratio, we develop an EFG method with DRK interpolants based on the Galerkin weak formulation of the CCST in this paper.

## 2. DRK Interpolants

In this paper, we use the DRK interpolant to model variations in each field variable across the microscale shell thickness. The relevant derivation of the DRK interpolant [47] is briefly outlined as follows:

There are $n_{p}$ discrete sampling nodes placed at natural coordinates, $\xi =\xi_{1},\;\xi_{2},\;\cdots ,\;\xi_{n_{p}}$, and their values in the natural coordinate system range from −1 to 1. The DRK interpolant (i.e., $f^{a}(\xi )$) used for simulating each field variable is expressed as follows:

(1) $$f^{a}(\xi )=\sum_{l=1}^{n_{p}}N_{l}(\xi )\;f_{l}=\sum_{l=1}^{n_{p}}\left[\phi_{l}(\xi )+\psi_{l}(\xi )\right]\;f_{l},$$

where $N_{l}\left(\xi \right)$ (*l* = 1, 2, …, $n_{p}$) denotes the DRK interpolant’s shape functions at the reference sampling point $\xi =\xi_{l}$; $f_{l}$ represents the nodal value of $f^{a}(\xi )$ at $\xi =\xi_{l}$. $\psi_{l}\left(\xi \right)$ is the selected primitive function satisfying the Kronecker delta properties, and $\phi_{l}\left(\xi \right)$ is the enrichment function determined by imposing the *n*th-order reproducing conditions. In our DRK meshless method, the enrichment function is expressed as follows:

(2) $$\phi_{l}(\xi )=w_{a}(\xi -\xi_{l})P^{T}(\xi -\xi_{l})b_{0}\left(\xi \right),$$

where $b_{0}\left(\xi \right)$ and $w_{a}\left(\xi -\xi_{l}\right)$ denote the undetermined function vector and a Gaussian function, respectively, and the transport of the base function set, $P^{T}\left(\xi -\xi_{l}\right)$, is provided as follows:

(3) $$P^{T}\left(\xi -\xi_{l}\right)=\left\{1\;\left(\xi -\xi_{l}\right)\;{\left(\xi -\xi_{l}\right)}^{2}\;\cdots \;{\left(\xi -\xi_{l}\right)}^{n}\right\}.$$

The undetermined function vector $b_{0}\left(\xi \right)$ can be determined by employing (*n* + 1) reproducing conditions as follows:

(4) $$\sum_{l=1}^{n_{p}}\left[\phi_{l}(\xi )+\psi_{l}(\xi )\right]\xi_{l}^{m}=\xi^{m},$$

where *m* is counted from 1 to *n*.

The undetermined function vector $b_{0}\left(\xi \right)$ can be obtained by substituting the enrichment functions into the reproducing condition (3) and expressed as follows:

(5) $$b_{0}\left(\xi \right)=A_{0}^{-1}(\xi )\left[P\left(0\right)-\sum_{l=1}^{n_{p}}P\left(\xi -\xi_{l}\right)\psi_{l}\left(\xi \right)\right],$$

where $A_{0}(\xi )=\sum_{l=1}^{n_{p}}\left[P\left(\xi -\xi_{l}\right)w_{a}\left(\xi -\xi_{l}\right)P^{T}\left(\xi -\xi_{l}\right)\right]$.

The DRK interpolant’s shape functions can thus be determined by substituting Equation (5) into Equation (2) and expressed as follows:

(6) $$N_{l}\left(\xi \right)=\phi_{l}\left(\xi \right)+\psi_{l}\left(\xi \right),$$

where the subscript *l* is counted from l=1 to $l=n_{p}$.

The derivatives of the DRK interpolant, including first- and higher-order derivatives, are obtained using the differential reproducing conditions without relying on the traditional, time-consuming differential operation. The primitive and weight functions, along with their related derivations, are provided in Wang et al. [47].

## 3. Hamilton’s Principle

**Figure 1:**
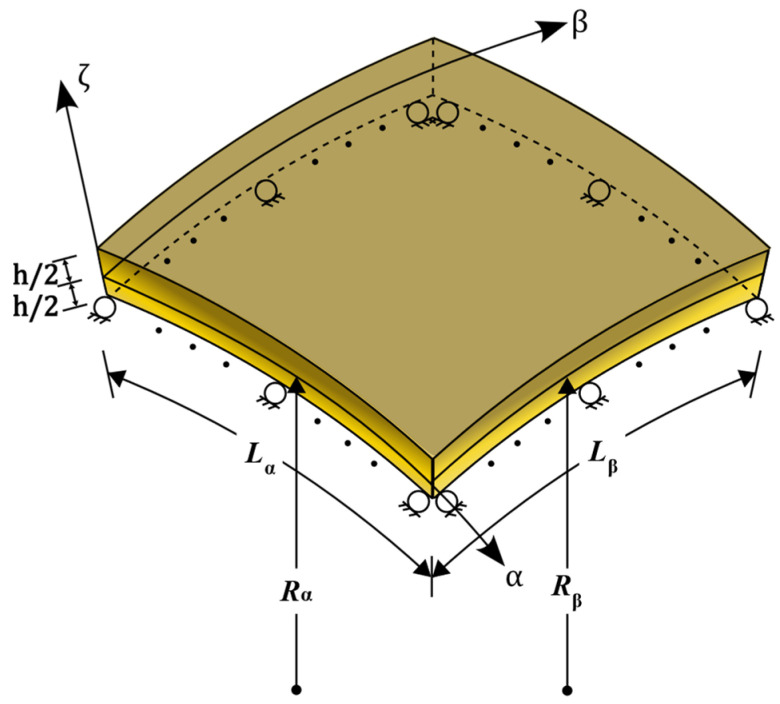
The schematic diagram of a typical FG doubly curved microscale shell under fully simply supported conditions. FG: Functionally graded.

This study examines the free vibration characteristics of an LC-/FG-MEE DC microscale shell under fully simply supported conditions and a uniform temperature change (see Figure 1). A DC shell coordinate system, α, β, and ζ, is positioned on the mid-surface of the microscale shell. The shell’s thickness is *h*, the mid-surface radii along α and β axes are $R_{\alpha }\;\mathrm{and}\;R_{\beta }$, and the in-surface dimensions along α and β axes are $L_{\alpha }\;\mathrm{and}\;L_{\beta }$.

### 3.1. Galerkin Weak Formulation

We derive the Galerkin weak formulation to solve this issue using Hamilton’s principle [48]. The energy functional of the microscale shell subjected to a uniform temperature change is provided as follows:

(7) $$I_{R}=\int_{t_{1}}^{t_{2}}\left(V-U_{s}+W\right)dt,$$

where *V* represents the kinetic energy functional of the microscale shell, $U_{s}$ denotes the strain energy functional of the microscale shell, and *W* is the work done by the thermally induced initial stresses. These energy functionals and work are expressed as follows:

(8) $$V=\int_{-h/2}^{h/2}\iint_{A}\left(\rho /2\right)\left[{\left(u_{\alpha ,t}\right)}^{2}+{\left(u_{\beta ,t}\right)}^{2}+{\left(u_{\zeta ,t}\right)}^{2}\right]\gamma_{\alpha }\;\gamma_{\beta }\;d\alpha \;d\beta \;d\zeta ,$$

(9) $$\begin{array}{ll} U_{s}=\int_{-h/2}^{h/2}\iint_{A}\left(1/2\right) & \left[\sigma_{\left(\alpha \alpha \right)}\varepsilon_{\alpha \alpha }+\sigma_{\left(\beta \beta \right)}\varepsilon_{\beta \beta }+\sigma_{\left(\zeta \zeta \right)}\varepsilon_{\zeta \zeta }\right.+\sigma_{\left(\alpha \zeta \right)}\gamma_{\alpha \zeta }+\sigma_{\left(\beta \zeta \right)}\gamma_{\beta \zeta }+\sigma_{\left(\alpha \beta \right)}\gamma_{\alpha \beta }\\ & -2\mu_{\alpha }\kappa_{\alpha }-2\mu_{\beta }\kappa_{\beta }-2\mu_{\zeta }\kappa_{\zeta }-D_{\alpha }E_{\alpha }-D_{\beta }E_{\beta }-D_{\zeta }E_{\zeta }\\ & \left.-B_{\alpha }H_{\alpha }-B_{\beta }H_{\beta }-B_{\zeta }H_{\zeta }\right]\gamma_{\alpha }\gamma_{\beta }d\alpha d\beta d\zeta , \end{array}$$

(10) $$W=\left(\Delta T\right)\int_{-h/2}^{h/2}\iint_{A}\left[c_{\alpha 1}\varepsilon_{\alpha \alpha }^{nl}+c_{\alpha 2}\varepsilon_{\beta \beta }^{nl}\right]\gamma_{\alpha }\gamma_{\beta }d\alpha d\beta d\zeta ,$$

where ρ denotes the mass density, *A* is the shell domain on the αβ-surface, and *t* is the time variable; $u_{i}$ represents the displacement tensors, and $\sigma_{\left(ij\right)}\;\mathrm{and}\;\varepsilon_{kl}$ are the symmetric parts of the force-stress tensor and strain tensor, respectively; $\mu_{i}\;\mathrm{and}\;\kappa_{i}$ represent the couple stress and the skew-symmetric part of the curvature tensors, *D_i_* and $B_{i}$ are the electric and magnetic flux tensors, and *E_i_* and *H_i_* are the electric and magnetic potential tensors; $\gamma_{\alpha }\;\mathrm{and}\;\gamma_{\beta }$ represent the scale factor for the DC shell coordinate system and are provided as $\gamma_{\alpha }=1+\left(\zeta /R_{\alpha }\right)$ and $\gamma_{\beta }=1+\left(\zeta /R_{\beta }\right)$; $c_{\alpha 1}\;\mathrm{and}\;c_{\alpha 2}$ represent the coupling force-stress and temperature change coefficients and are provided as $c_{\alpha 1}=c_{11}\alpha_{1}+c_{12}\alpha_{2}+c_{13}\alpha_{3}$, and $c_{\alpha 2}=c_{21}\alpha_{1}+c_{22}\alpha_{2}+c_{23}\alpha_{3}$, in which $c_{ij}\;\mathrm{and}\;\alpha_{i}$ represent the elastic and thermal expansion coefficients; and $\varepsilon_{\alpha \alpha }^{nl}\;\mathrm{and}\;\varepsilon_{\beta \beta }^{nl}$ are the full nonlinear strains and are provided as follows:

(11) $$\varepsilon_{\alpha \alpha }^{nl}=\left[{\left(u_{\zeta }/R_{\alpha }\right)}^{2}+{\left(u_{\zeta ,\alpha }\right)}^{2}-2u_{\zeta ,\alpha }\left(u_{\alpha }/R_{\alpha }\right)+{\left(u_{\alpha }/R_{\alpha }\right)}^{2}\right]/\left(2\gamma_{\alpha }^{2}\right),$$

(12) $$\varepsilon_{\beta \beta }^{nl}=\left[{\left(u_{\zeta }/R_{\beta }\right)}^{2}+{\left(u_{\zeta ,\beta }\right)}^{2}-2u_{\zeta ,\beta }\left(u_{\beta }/R_{\beta }\right)+{\left(u_{\beta }/R_{\beta }\right)}^{2}\right]/\left(2\gamma_{\beta }^{2}\right).$$

This study assumes a uniform temperature change and does not perform a heat conduction analysis because the temperature variations arise from the difference between the manufacturing and working environments. The shell dimensions considered are very small, approaching the micron scale, so the temperature should quickly reach a uniform steady state after the transient phase dissipates.

We choose the displacement components as the primary field variables subjected to variation; the other variables, including strain, rotation, and curvature components, are the dependent field variables, and their relationship is expressed as follows:

The strain-displacement relationship is given as follows [49]:

(13) $$\varepsilon_{\alpha \alpha }=\left[u_{\alpha ,\alpha }+\left(u_{\zeta }/R_{\alpha }\right)\right]/\gamma_{\alpha },$$

(14) $$\varepsilon_{\beta \beta }=\left[u_{\beta ,\beta }+\left(u_{\zeta }/R_{\beta }\right)\right]/\gamma_{\beta },$$

(15) $$\varepsilon_{\zeta \zeta }=u_{\zeta ,\zeta },$$

(16) $$\gamma_{\beta \zeta }=u_{\beta ,\zeta }-u_{\beta }/\left(\gamma_{\beta }R_{\beta }\right)+\left(u_{\zeta ,\beta }\right)/\gamma_{\beta },$$

(17) $$\gamma_{\alpha \zeta }=u_{\alpha ,\zeta }-u_{\alpha }/\left(\gamma_{\alpha }R_{\alpha }\right)+\left(u_{\zeta ,\alpha }\right)/\gamma_{\alpha },$$

(18) $$\gamma_{\alpha \beta }=\left(u_{\alpha ,\beta }/\gamma_{\beta }\right)+\left(u_{\beta ,\alpha }/\gamma_{\alpha }\right).$$

The rotation-displacement relationship is given as follows [49]

(19) $$\theta_{\alpha }=-\left(u_{\beta ,\zeta }/2\right)-u_{\beta }/\left(2R_{\beta }\gamma_{\beta }\right)+u_{\zeta ,\beta }/\left(2\gamma_{\beta }\right),$$

(20) $$\theta_{\beta }=\left(u_{\alpha ,\zeta }/2\right)+u_{\alpha }/\left(2R_{\alpha }\gamma_{\alpha }\right)-u_{\zeta ,\alpha }/\left(2\gamma_{\alpha }\right),$$

(21) $$\theta_{\zeta }=-u_{\alpha ,\beta }/\left(2\gamma_{\beta }\right)+u_{\beta ,\alpha }/\left(2\gamma_{\alpha }\right).$$

The skew-symmetric parts of the curvature-rotation relationship are given as follows [49]:

(22) $$\begin{array}{ll} \kappa_{\alpha } & =\left\{\left[-1/\left(4\gamma_{\beta }^{2}\right)\right]\right.\partial_{\beta \beta }-\left(1/4\right)\partial_{\zeta \zeta }-\left[1/\left(4R_{\alpha }\gamma_{\alpha }\right)+1/\left(4R_{\beta }\gamma_{\beta }\right)\right]\partial_{\zeta }+\left[1/\left(4R_{\alpha }^{2}\gamma_{\alpha }^{2}\right)\right.-1/\left.\left.\left(4R_{\alpha }R_{\beta }\gamma_{\alpha }\gamma_{\beta }\right)\right]\right\}u_{\alpha }\\ & +\left[1/\left(4\gamma_{\alpha }\gamma_{\beta }\right)\right]u_{\beta ,\alpha \beta }+\left\{\left[-1/\left(4R_{\alpha }\gamma_{\alpha }^{2}\right)+1/\left(4R_{\beta }\gamma_{\alpha }\gamma_{\beta }\right)\right]\partial_{\alpha }+\left[1/\left(4\gamma_{\alpha }\right)\right]\partial_{\alpha \zeta }\right\}u_{\zeta }, \end{array}$$

(23) $$\begin{array}{ll} \kappa_{\beta } & =\left[1/\left(4\gamma_{\alpha }\gamma_{\beta }\right)\right]u_{\alpha ,\alpha \beta }+\left\{-1/\left(4\gamma_{\alpha }^{2}\right)\partial_{\alpha \alpha }-\left(1/4\right)\partial_{\zeta \zeta }\right.-\left[1/\left(4R_{\alpha }\gamma_{\alpha }\right)\right.\left.+1/\left(4R_{\beta }\gamma_{\beta }\right)\right]\partial_{\zeta }\\ & +\left.\left[1/\left(4R_{\beta }^{2}\gamma_{\beta }^{2}\right)-1/\left(4R_{\alpha }R_{\beta }\gamma_{\alpha }\gamma_{\beta }\right)\right]\right\}u_{\beta }+\left\{\left[-1/\left(4R_{\beta }\gamma_{\beta }^{2}\right)+1/\left(4R_{\alpha }\gamma_{\alpha }\gamma_{\beta }\right)\right]\partial_{\beta }+\left[1/\left(4\gamma_{\beta }\right)\right]\partial_{\beta \zeta }\right\}u_{\zeta }, \end{array}$$

(24) $$\begin{array}{ll} \kappa_{\zeta } & =\left\{\left[1/\left(4R_{\alpha }\gamma_{\alpha }^{2}\right)\right]\partial_{\alpha }+\left[1/\left(4\gamma_{\alpha }\right)\right]\partial_{\alpha \zeta }\right\}u_{\alpha }+\left\{\left[1/\left(4R_{\beta }\gamma_{\beta }^{2}\right)\right]\partial_{\beta }+\left[1/\left(4\gamma_{\beta }\right)\right]\partial_{\beta \zeta }\right\}u_{\beta }\\ & -\left\{\left[1/\left(4\gamma_{\alpha }^{2}\right)\right]\partial_{\alpha \alpha }+\left[1/\left(4\gamma_{\beta }^{2}\right)\right]\partial_{\beta \beta }\right\}u_{\zeta }. \end{array}$$

The constitutive equations suitable for an orthotropic material are given as follows:

(25) $$\left\{\begin{array}{c} \sigma_{\left(\alpha \alpha \right)}\\ \sigma_{\left(\beta \beta \right)}\\ \sigma_{\left(\zeta \zeta \right)}\\ \sigma_{\left(\beta \zeta \right)}\\ \sigma_{\left(\alpha \zeta \right)}\\ \sigma_{\left(\alpha \beta \right)} \end{array}\right\}=\left[\begin{array}{cccccc} c_{11} & c_{12} & c_{13} & 0 & 0 & 0\\ c_{12} & c_{22} & c_{23} & 0 & 0 & 0\\ c_{13} & c_{23} & c_{33} & 0 & 0 & 0\\ 0 & 0 & 0 & c_{44} & 0 & 0\\ 0 & 0 & 0 & 0 & c_{55} & 0\\ 0 & 0 & 0 & 0 & 0 & c_{66} \end{array}\right]\left\{\begin{array}{c} \varepsilon_{\alpha \alpha }\\ \varepsilon_{\beta \beta }\\ \varepsilon_{\zeta \zeta }\\ \gamma_{\beta \zeta }\\ \gamma_{\alpha \zeta }\\ \gamma_{\alpha \beta } \end{array}\right\}-\left[\begin{array}{ccc} 0 & 0 & e_{31}\\ 0 & 0 & e_{32}\\ 0 & 0 & e_{33}\\ 0 & e_{24} & 0\\ e_{15} & 0 & 0\\ 0 & 0 & 0 \end{array}\right]\left\{\begin{array}{c} E_{\alpha }\\ E_{\beta }\\ E_{\zeta } \end{array}\right\}-\left[\begin{array}{ccc} 0 & 0 & q_{31}\\ 0 & 0 & q_{32}\\ 0 & 0 & q_{33}\\ 0 & q_{24} & 0\\ q_{15} & 0 & 0\\ 0 & 0 & 0 \end{array}\right]\left\{\begin{array}{c} H_{\alpha }\\ H_{\beta }\\ H_{\zeta } \end{array}\right\}-\left\{\begin{array}{c} c_{\alpha 1}\\ c_{\alpha 2}\\ c_{\alpha 3}\\ 0\\ 0\\ 0 \end{array}\right\}\left(\Delta T\right),$$

(26) $$\left\{\begin{array}{c} D_{\alpha }\\ D_{\beta }\\ D_{\zeta } \end{array}\right\}=\left[\begin{array}{cccccc} 0 & 0 & 0 & 0 & e_{15} & 0\\ 0 & 0 & 0 & e_{24} & 0 & 0\\ e_{31} & e_{32} & e_{33} & 0 & 0 & 0 \end{array}\right]\left\{\begin{array}{c} \varepsilon_{\alpha \alpha }\\ \varepsilon_{\beta \beta }\\ \varepsilon_{\zeta \zeta }\\ \gamma_{\beta \zeta }\\ \gamma_{\alpha \zeta }\\ \gamma_{\alpha \beta } \end{array}\right\}+\left[\begin{array}{ccc} \eta_{11} & 0 & 0\\ 0 & \eta_{22} & 0\\ 0 & 0 & \eta_{33} \end{array}\right]\left\{\begin{array}{c} E_{\alpha }\\ E_{\beta }\\ E_{\zeta } \end{array}\right\}+\left[\begin{array}{ccc} d_{11} & 0 & 0\\ 0 & d_{22} & 0\\ 0 & 0 & d_{33} \end{array}\right]\left\{\begin{array}{c} H_{\alpha }\\ H_{\beta }\\ H_{\zeta } \end{array}\right\}-\left\{\begin{array}{c} 0\\ 0\\ e_{\alpha 3} \end{array}\right\}\left(\Delta T\right),$$

(27) $$\left\{\begin{array}{c} B_{\alpha }\\ B_{\beta }\\ B_{\zeta } \end{array}\right\}=\left[\begin{array}{cccccc} 0 & 0 & 0 & 0 & q_{15} & 0\\ 0 & 0 & 0 & q_{24} & 0 & 0\\ q_{31} & q_{32} & q_{33} & 0 & 0 & 0 \end{array}\right]\left\{\begin{array}{c} \varepsilon_{\alpha \alpha }\\ \varepsilon_{\beta \beta }\\ \varepsilon_{\zeta \zeta }\\ \gamma_{\beta \zeta }\\ \gamma_{\alpha \zeta }\\ \gamma_{\alpha \beta } \end{array}\right\}+\left[\begin{array}{ccc} d_{11} & 0 & 0\\ 0 & d_{22} & 0\\ 0 & 0 & d_{33} \end{array}\right]\left\{\begin{array}{c} E_{\alpha }\\ E_{\beta }\\ E_{\zeta } \end{array}\right\}+\left[\begin{array}{ccc} \beta_{11} & 0 & 0\\ 0 & \beta_{22} & 0\\ 0 & 0 & \beta_{33} \end{array}\right]\left\{\begin{array}{c} H_{\alpha }\\ H_{\beta }\\ H_{\zeta } \end{array}\right\}-\left\{\begin{array}{c} 0\\ 0\\ q_{\alpha 3} \end{array}\right\}\left(\Delta T\right),$$

where $c_{ij},\;e_{ij},\;\mathrm{and}\;q_{ij}$ represent the elastic, piezoelectric, and magnetoelastic coefficients; $\eta_{kk},\;d_{kk},\;\mathrm{and}\;\beta_{kk}$ represent the dielectric permeability, piezomagnetic, and magnetic permeability coefficients; $e_{\alpha 3}$ and $q_{\alpha 3}$ represent the coupling electric flux and temperature change and coupling magnetic flux and temperature change coefficients, which are given as $e_{\alpha 3}=e_{31}\alpha_{1}+e_{32}\alpha_{2}+e_{33}\alpha_{3}$ and $q_{\alpha 3}=q_{31}\alpha_{1}+q_{32}\alpha_{2}+q_{33}\alpha_{3}$, respectively; *E_i_* and $H_{i}$ represent the electric and magnetic field variables and are provided as $E_{i}^{\left(m\right)}=-\Phi_{,i}$ and $H_{i}=-\Psi_{,i}$ in which Φ and Ψ represent the electric and magnetic potential variables.

The couple stress tensor-skew-symmetric parts of the curvature tensor relationship is expressed as follows [49]:

(28) $$\left\{\begin{array}{c} \mu_{\alpha }\\ \mu_{\beta }\\ \mu_{\zeta } \end{array}\right\}=-\left(1/2\right)\left[\begin{array}{ccc} b_{11} & 0 & 0\\ 0 & b_{22} & 0\\ 0 & 0 & b_{33} \end{array}\right]\left\{\begin{array}{c} \kappa_{\alpha }\\ \kappa_{\beta }\\ \kappa_{\zeta } \end{array}\right\},$$

where $b_{kk}$ represents the coupling couple stress and the skew-symmetric parts of the curvature coefficient. For an isotropic material, $b_{kk}=4G_{ji}l^{2}$, in which *G_ji_* represents the shear modulus of the *ji*-surface and *l* represents the material length-scale parameter.

By taking the first-order variation of the energy functional expressed in Equation (7) and setting it to zero, the authors derive the Galerkin weak formulation of the 3D CCST for analyzing static buckling and free vibration characteristics of an FG-DC microscale shell in a temperature environment as follows:

(29) $$\begin{array}{l} \delta I_{R}=0\\ \Rightarrow \int_{t_{1}}^{t_{2}}\left\{\delta V-\delta U_{s}+\delta W\right\}dt=0, \end{array}$$

where

(30) $$\int_{t_{1}}^{t_{2}}\delta Vdt=-\int_{t_{1}}^{t_{2}}\left\{\int_{-h/2}^{h/2}\iint_{\Omega }\rho \left[{\left(\delta u\right)}^{T}u_{,tt}+\left(\delta u_{\zeta }\right)u_{\zeta ,tt}\right]\gamma_{\alpha }\gamma_{\beta }d\alpha d\beta d\zeta \right\}dt,$$

(31) $$\begin{array}{ll} \int_{t_{1}}^{t_{2}}\delta U_{s}dt & =\int_{t_{1}}^{t_{2}}\left\{\int_{-h/2}^{h/2}\iint_{A}\left[\delta {\left(\varepsilon_{p}\right)}^{T}\sigma_{p}+\delta {\left(\varepsilon_{s}\right)}^{T}\sigma_{s}+\delta \varepsilon_{\zeta \zeta }\sigma_{\zeta \zeta }-2\delta {\left(\kappa \right)}^{T}\mu \right.\right.\\ & -\delta {\left(E_{p}\right)}^{T}D_{p}-\delta E_{\zeta }D_{\zeta }\left.\left.-\delta {\left(H_{p}\right)}^{T}B_{p}-\delta H_{\zeta }B_{\zeta }\right]\gamma_{\alpha }\gamma_{\beta }d\alpha d\beta d\zeta \right\}dt, \end{array}$$

(32) $$\begin{array}{c} \int_{t_{1}}^{t_{2}}\delta Wdt=\left(\Delta T\right)\int_{t_{1}}^{t_{2}}\left\{\int_{-h/2}^{h/2}\iint_{A}\left\{\delta u_{\alpha }\left[-c_{\alpha 1}u_{\zeta ,\alpha }/\left(\gamma_{\alpha }^{2}R_{\alpha }\right)+c_{\alpha 1}u_{\alpha }/\left(\gamma_{\alpha }^{2}R_{\alpha }^{2}\right)\right]\right.\right.\\ +\delta u_{\beta }\left[-c_{\alpha 2}u_{\zeta ,\beta }/\left(\gamma_{\beta }^{2}R_{\beta }\right)+c_{\alpha 2}u_{\beta }/\left(\gamma_{\beta }^{2}R_{\beta }^{2}\right)\right]+\delta u_{\zeta }\left[c_{\alpha 1}u_{\zeta }/\left(\gamma_{\alpha }^{2}R_{\alpha }^{2}\right)\right.\\ \left.+c_{\alpha 2}u_{\zeta }/\left(\gamma_{\beta }^{2}R_{\beta }^{2}\right)\right]+\delta u_{\zeta ,\alpha }\left[c_{\alpha 1}u_{\zeta ,\alpha }/\left(\gamma_{\alpha }^{2}\right)-c_{\alpha 1}u_{\alpha }/\left(\gamma_{\alpha }^{2}R_{\alpha }\right)\right]\\ +\left.\delta u_{\zeta ,\beta }\left[c_{\alpha 2}u_{\zeta ,\beta }/\left(\gamma_{\beta }^{2}\right)-c_{\alpha 2}u_{\beta }/\left(\gamma_{\beta }^{2}R_{\beta }\right)\right]\right\}dt. \end{array}$$

Additionally, various field variables shown in Equations (30)–(32) are expressed as follows:

(33) $$u={\left\{\begin{array}{cc} u_{\alpha } & u_{\beta } \end{array}\right\}}^{T},$$

(34) $$\varepsilon_{p}={\left\{\begin{array}{ccc} \varepsilon_{\alpha \alpha } & \varepsilon_{\beta \beta } & \gamma_{\alpha \beta } \end{array}\right\}}^{T}=B_{1}u+B_{2}u_{\zeta },$$

(35) $$\varepsilon_{s}={\left\{\begin{array}{cc} \gamma_{\alpha \zeta } & \gamma_{\beta \zeta } \end{array}\right\}}^{T}=B_{3}u+B_{4}u_{\zeta },$$

(36) $$\varepsilon_{\zeta \zeta }=B_{5}u_{\zeta },$$

(37) $$\kappa ={\left\{\begin{array}{ccc} \kappa_{\alpha } & \kappa_{\beta } & \kappa_{\zeta } \end{array}\right\}}^{T}=B_{6}u+B_{7}u_{\zeta },$$

(38) $$E_{p}={\left\{\begin{array}{cc} E_{\alpha } & E_{\beta } \end{array}\right\}}^{T}=B_{8}\Phi ,$$

(39) $$E_{\zeta }=-\Phi_{,\zeta },$$

(40) $$H_{p}={\left\{\begin{array}{cc} H_{\alpha } & H_{\beta } \end{array}\right\}}^{T}=B_{8}\Psi ,$$

(41) $$H_{\zeta }=-\Psi_{,\zeta },$$

(42) $$\sigma_{p}={\left\{\begin{array}{ccc} \sigma_{\left(\alpha \alpha \right)} & \sigma_{\left(\beta \beta \right)} & \sigma_{\left(\alpha \beta \right)} \end{array}\right\}}^{T}=Q_{p}\varepsilon_{p}+Q_{\zeta }\varepsilon_{\zeta \zeta }+Q_{1}E_{\zeta }+Q_{2}H_{\zeta }-Q_{3}\Delta T,$$

(43) $$\sigma_{s}={\left\{\begin{array}{cc} \sigma_{\left(\beta \zeta \right)} & \sigma_{\left(\alpha \zeta \right)} \end{array}\right\}}^{T}=Q_{s}\varepsilon_{s}+Q_{e}E_{p}+Q_{q}H_{p},$$

(44) $$\sigma_{\zeta \zeta }={\left(Q_{\zeta }\right)}^{T}\varepsilon_{p}+c_{33}\varepsilon_{\zeta \zeta }+e_{33}E_{\zeta }+q_{33}H_{\zeta }-c_{\alpha 3}\Delta T,$$

(45) $$\mu ={\left\{\begin{array}{ccc} \mu_{\alpha } & \mu_{\beta } & \mu_{\zeta } \end{array}\right\}}^{T}=-Q_{c}\kappa ,$$

(46) $$D_{p}={\left\{\begin{array}{cc} D_{\alpha } & D_{\beta } \end{array}\right\}}^{T}=Q_{e}\varepsilon_{s}+Q_{\eta }E_{p}+Q_{d}H_{p},$$

(47) $$D_{\zeta }={\left(Q_{1}\right)}^{T}\varepsilon_{p}+e_{33}\varepsilon_{\zeta \zeta }+\eta_{33}E_{\zeta }+d_{33}H_{\zeta }-e_{\alpha 3}\Delta T,$$

where relevant matrices and vectors shown in Equations (33)–(47) are provided in Appendix A.

The Galerkin weak formulation can thus be derived by substituting Equations (30)–(32) into Equation (29).

### 3.2. System Equations

Incorporating the DRK interpolant given in Equation (3) into the Galerkin weak formulation derived above, we develop the EFG method as follows.

In the case of interest, the tractions on the microscale shell’s top and bottom surfaces are free, and the edge conditions of the microscale shell are simply supported. These surface and edge conditions are written as follows:

The conditions of the microscale shell’s top and bottom surfaces are as follows:

(48) $$\mathrm{Case}\;1:\;\sigma_{\zeta \alpha }=\sigma_{\zeta \beta }=\sigma_{\zeta \zeta }=\mu_{\zeta \alpha }=\mu_{\zeta \beta }=\Phi =\Psi =0\;\;\;\mathrm{on}\;\mathrm{the}\;\mathrm{surfaces}\;\mathrm{at}\;\zeta =\pm \left(h/2\right),$$

(49) $$\mathrm{Case}\;2:\;\sigma_{\zeta \alpha }=\sigma_{\zeta \beta }=\sigma_{\zeta \zeta }=\mu_{\zeta \alpha }=\mu_{\zeta \beta }=D_{\zeta }=B_{\zeta }=0\;\;\;\mathrm{on}\;\mathrm{the}\;\mathrm{surfaces}\;\mathrm{at}\;\zeta =\pm \left(h/2\right).$$

The edge conditions of the microscale shell are provided as follows:

(50) $$u_{\beta }^{\left(m\right)}=u_{\zeta }^{\left(m\right)}=\sigma_{\alpha \alpha }^{\left(m\right)}=\mu_{\alpha \beta }^{\left(m\right)}=\mu_{\alpha \zeta }^{\left(m\right)}=\Phi^{\left(m\right)}=\psi^{\left(m\right)}=0\;\;\;\;\;\mathrm{at}\;\alpha =0\;\mathrm{and}\;\alpha =a_{\alpha },$$

(51) $$u_{\alpha }^{\left(m\right)}=u_{\zeta }^{\left(m\right)}=\sigma_{\beta \beta }^{\left(m\right)}=\mu_{\beta \alpha }^{\left(m\right)}=\mu_{\beta \zeta }^{\left(m\right)}=\Phi^{\left(m\right)}=\psi^{\left(m\right)}=0\;\;\;\;\;\mathrm{at}\;\beta =0\;\mathrm{and}\;\beta =a_{\beta },$$

where $m=1,\;2,\cdots ,\;n_{l}$.

By satisfying the edge conditions (i.e., Equations (50) and (51)) in prior, we express each layer’s displacement variables as follows:

(52) $$u_{\alpha }=\sum_{\hat{m}=1}^{\infty }\sum_{\hat{n}=1}^{\infty }u_{\hat{m}\hat{n}}(\zeta )\cos\tilde{m}\alpha \;\sin\tilde{n}\beta \;e^{i\omega t},$$

(53) $$u_{\beta }=\sum_{\hat{m}=1}^{\infty }\sum_{\hat{n}=1}^{\infty }v_{\hat{m}\hat{n}}(\zeta )\sin\tilde{m}\alpha \;\cos\tilde{n}\beta \;e^{i\omega t},$$

(54) $$u_{\zeta }=\sum_{\hat{m}=1}^{\infty }\sum_{\hat{n}=1}^{\infty }w_{\hat{m}\hat{n}}(\zeta )\sin\tilde{m}\alpha \;\sin\tilde{n}\beta \;e^{i\omega t},$$

(55) $$\Phi =\sum_{\hat{m}=1}^{\infty }\sum_{\hat{n}=1}^{\infty }\phi_{\hat{m}\hat{n}}(\zeta )\sin\tilde{m}\alpha \;\sin\tilde{n}\beta \;e^{i\omega t},$$

(56) $$\Psi =\sum_{\hat{m}=1}^{\infty }\sum_{\hat{n}=1}^{\infty }\psi_{\hat{m}\hat{n}}(\zeta )\sin\tilde{m}\alpha \;\sin\tilde{n}\beta \;e^{i\omega t},$$

where the symbols $\hat{m}$ and $\hat{n}$ are positive integers representing the half-wave numbers in the α and β directions, and $\tilde{m}=\hat{m}\pi /a_{\alpha }$ and $\tilde{n}=\hat{n}\pi /a_{\beta }$.

In the EFG method, we interpolate the primary variables $u_{\hat{m}\hat{n}},\;$$v_{\hat{m}\hat{n}},\;w_{\hat{m}\hat{n}},\;\phi_{\hat{m}\hat{n}},\;$$\mathrm{and}\;\psi_{\hat{m}\hat{n}}$ with the DRK interpolant given in Section 2, such that

(57) $$F_{\hat{m}\hat{n}}=\sum_{l=1}^{n_{p}}\left(\phi_{l}+\psi_{l}\right){\left(F_{\hat{m}\hat{n}}\right)}_{l}=\sum_{l=1}^{n_{p}}N_{l}{\left(F_{\hat{m}\hat{n}}\right)}_{l},$$

where *F* represents *u*, *v*, *w*, ϕ, and ψ, and ${\left(F_{\hat{m}\hat{n}}\right)}_{l}=F_{\hat{m}\hat{n}}\left(\zeta =\zeta_{l}\right).$

Substituting Equations (30)–(32) and (52)–(57) into Equation (29) yields the layer element equations of the microscale shell as follows:

(58) $$\sum_{i=1}^{n_{p}}\sum_{j=1}^{n_{p}}\left(\left[\begin{array}{ccccc} k_{11} & k_{12} & k_{13} & k_{14} & k_{15}\\ k_{21} & k_{22} & k_{23} & k_{24} & k_{25}\\ k_{31} & k_{32} & k_{33} & k_{34} & k_{35}\\ k_{41} & k_{42} & k_{43} & k_{44} & k_{45}\\ k_{51} & k_{52} & k_{53} & k_{54} & k_{55} \end{array}\right]-\omega^{2}\left[\begin{array}{ccccc} m_{11} & 0 & 0 & 0 & 0\\ 0 & m_{22} & 0 & 0 & 0\\ 0 & 0 & m_{33} & 0 & 0\\ 0 & 0 & 0 & 0 & 0\\ 0 & 0 & 0 & 0 & 0 \end{array}\right]-\Delta T\left[\begin{array}{ccccc} g_{11} & 0 & g_{13} & 0 & 0\\ 0 & g_{22} & g_{23} & 0 & 0\\ g_{31} & g_{32} & g_{33} & 0 & 0\\ 0 & 0 & 0 & 0 & 0\\ 0 & 0 & 0 & 0 & 0 \end{array}\right]\right)\left\{\begin{array}{c} {\left(u_{\hat{m}\hat{n}}\right)}_{j}\\ {\left(v_{\hat{m}\hat{n}}\right)}_{j}\\ {\left(w_{\hat{m}\hat{n}}\right)}_{j}\\ {\left(\phi_{\hat{m}\hat{n}}\right)}_{j}\\ {\left(\psi_{\hat{m}\hat{n}}\right)}_{j} \end{array}\right\}=0,$$

where *k_ij_*, *m_kk_*, and *g_ij_* represent the stiffness, mass, and geometric stiffness matrix coefficients, whose detailed expressions are provided in Appendix B.

By assembling the equations of each layer element, we can obtain the corresponding system of equations, which consists of $5n_{p}$ simultaneous algebraic equations expressed in terms of $5n_{p}$ nodal displacement components, rewritten as follows:

(59) $$\left(\left[\begin{array}{cc} K_{I\;I} & K_{I\;II}\\ K_{II\;I} & K_{II\;II} \end{array}\right]-\omega^{2}\left[\begin{array}{cc} M_{I\;I} & 0\\ 0 & 0 \end{array}\right]-\Delta T\left[\begin{array}{cc} G_{I\;I} & 0\\ 0 & 0 \end{array}\right]\right)\left\{\begin{array}{c} X_{I}\\ X_{II} \end{array}\right\}=0,$$

where $X_{I}={\left\{{\left(u_{\hat{m}\hat{n}}\right)}_{j}\;{\left(v_{\hat{m}\hat{n}}\right)}_{j}\;{\left(w_{\hat{m}\hat{n}}\right)}_{j}\;\right\}}^{T}$ and $X_{II}={\left\{\begin{array}{cc} {\left(\phi_{\hat{m}\hat{n}}\right)}_{j} & {\left(\psi_{\hat{m}\hat{n}}\right)}_{j} \end{array}\right\}}^{T}$, and *j* = 1, 2, …, $n_{p}$.

For the pure free vibration problem, we let ΔT=0, and the corresponding system equations are obtained as follows:

(60) $$\left(\left[\begin{array}{cc} K_{I\;I} & K_{I\;II}\\ K_{II\;I} & K_{II\;II} \end{array}\right]-\omega^{2}\left[\begin{array}{cc} M_{I\;I} & 0\\ 0 & 0 \end{array}\right]\right)\left\{\begin{array}{c} X_{I}\\ X_{II} \end{array}\right\}=0.$$

The natural frequency of the microscale shell can be determined by setting the determinant of the coefficient matrix of Equation (60) to zero as follows:

(61) $$\left|\left[\begin{array}{cc} K_{I\;I} & K_{I\;II}\\ K_{II\;I} & K_{II\;II} \end{array}\right]-\omega^{2}\left[\begin{array}{cc} M_{I\;I} & 0\\ 0 & 0 \end{array}\right]\right|=0.$$

For the free vibration problem considering a thermal environment, the corresponding system equations are derived as follows:

(62) $$\left(\left[\begin{array}{cc} {\hat{K}}_{I\;I} & K_{I\;II}\\ K_{II\;I} & K_{II\;II} \end{array}\right]-\omega^{2}\left[\begin{array}{cc} M_{I\;I} & 0\\ 0 & 0 \end{array}\right]\right)\left\{\begin{array}{c} X_{I}\\ X_{II} \end{array}\right\}=0,$$

where ${\hat{K}}_{I\;I}=K_{I\;I}-\Delta TG_{I\;I}.$

The natural frequency of the microscale shell subjected to a uniform temperature change can be determined by setting the determinant of the coefficient matrix of Equation (62) to zero as follows:

(63) $$\left|\left[\begin{array}{cc} {\hat{K}}_{I\;I} & K_{I\;II}\\ K_{II\;I} & K_{II\;II} \end{array}\right]-\omega^{2}\left[\begin{array}{cc} M_{I\;I} & 0\\ 0 & 0 \end{array}\right]\right|=0.$$

## 4. Numerical Examples

### 4.1. LC and FG-MEE Macro and Microscale Plates

This section examines the free vibration characteristics of simply supported, three-layered composite and FG-MEE macro- and microscale plates under two surface conditions (i.e., Cases 1 and 2). The three-layered plate consists of BaTiO_3_ (B) and CoFe_2_O_4_, (F) layers, with material properties provided in Table 1 [50,51].

Table 2 examines two different lay-ups (i.e., B/F/B and F/B/F). Table 3 presents the FG plate, composed of B and F materials with varying thicknesses, following a power-law distribution of the constituents’ volume fractions. The volume fractions of B and F are given as follows:

(64) $$\Gamma_{B}={\left[\left(1/2\right)+\left(z/h\right)\right]}^{\kappa_{p}}$$

(65) $$\Gamma_{F}=1-\Gamma_{B},$$

where $\kappa_{p}$ denotes the inhomogeneity index.

The effective material properties of the FG plate are estimated using a rule of mixtures [48] and are expressed as follows:

(66) $$P_{eff}=P_{B}\Gamma_{B}+P_{F}\Gamma_{F},$$

where *P* represents each material property.

In the macroscale plate cases, the geometric parameters are provided as $L_{\alpha }=L_{\beta }=1\;m,$ and h=0.3 m. In the microscale plate cases, the material length-scale parameter is provided as $l=17.6\times 10^{-6}\;m$, and the *l*/*h* ratios are 0.25, 0.5, and 1. For the three-layered plates, a dimensionless frequency, following Chen et al. [50], is defined as $\bar{\omega }=\omega L_{\alpha }\sqrt{\rho_{F}/{\left(c_{11}\right)}_{F}}$. For the FG plates, the dimensionless frequency, following Ramirez et al. [51], is defined as $\bar{\omega }=\omega {\left(L_{\alpha }\right)}^{2}\sqrt{\rho_{F}/{\left(c_{11}\right)}_{F}}/h$. The current EFG method for analyzing LC/FG-MEE DC microscale shells can be applied to analyze LC/FG-MEE macroscale plates by setting the material length-scale parameter and the inverse of the radii to zero (i.e., *l* = 0 and $1/R_{\alpha }=1/R_{\beta }=0$). When executing the EFG method, we select the highest order of the base functions as *n* = 5, and the size of the influence zone is $a_{l}=5.1\Delta \zeta ,$ where $\Delta \zeta =h/\left(n_{p}-1\right).$ The accuracy and convergence of the EFG method are validated using the 3D exact solutions obtained by Chen et al. [50] with the state-space method and Wu and Lu [52] with the modified Pagano method.

Table 2 and Table 3 display the results for the first five frequencies of the three-layered composite and FG MEE macro- and microscale plates. As shown in these tables, the solutions from the EFG method are accurate and converge rapidly. When *n_p_* = 31, the relative error between the current solutions and the 3D exact solutions [50,52] is less than 0.6%. The solutions obtained from the EFG method outperform those from the discrete-layer approach of Remirez et al. [51]. Moreover, the results indicate that increasing the material length-scale parameter increases the overall stiffness of the three-layered composite and the FG microplate, thereby raising their natural frequencies. Table 3 shows that the lowest frequency increases as the inhomogeneity index increases. This occurs because, as the inhomogeneity index increases, the volume fraction of the stiffer material, F, increases, thereby enhancing the overall stiffness of the FG plate and consequently its lowest frequency. Additionally, Table 3 demonstrates that the lowest frequency of the FG-MEE plate decreases as the plate becomes thinner, confirming that reducing the plate’s thickness lowers its overall stiffness and, in turn, its lowest frequency.

### 4.2. FG-MEE DC Microscale Shells

This section presents a parametric study to analyze how several key factors influence the free vibration characteristics of a simply supported FG-MEE DC microscale shell. These factors include the material length-scale parameter, the inhomogeneity index, the temperature change, the mid-surface radius ratio, and the length-to-thickness ratio. The microscale shell studied is made of B and F materials, with volume fractions of the constituents following a power-law distribution. The volume fractions of B and F materials, along with the estimation of the effective material properties, are provided in the same manner as in Equations (64)–(66). A dimensionless frequency parameter is also defined as $\bar{\omega }=\omega L_{\alpha }\sqrt{\rho_{F}/{\left(c_{11}\right)}_{F}}$.

**Figure 2:**
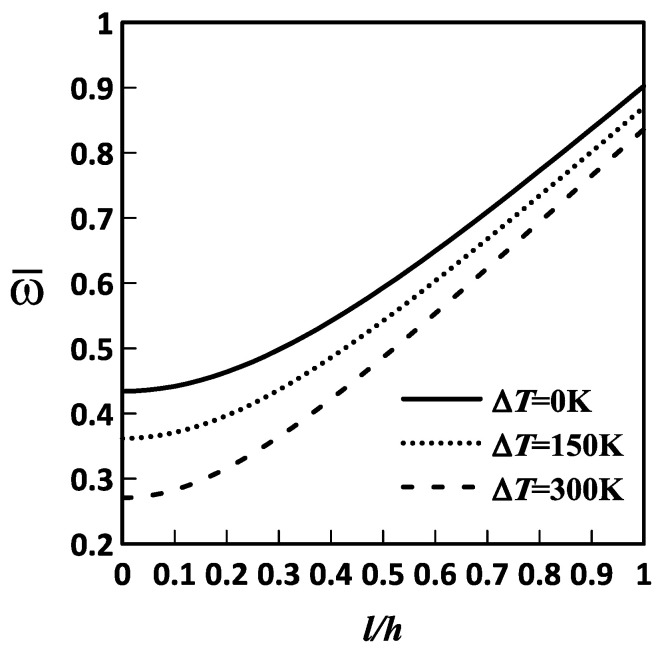
Variations in the lowest frequency of an FG-MEE DC microscale shell with the *l*/*h* ratio for ΔT= 0, 150, and 300 K. DC: Doubly curved; FG: Functionally graded; MEE: Magneto-electro-elastic.

Figure 2 shows the variations in the lowest frequency of the FG-MEE DC microscale shell with the *l*/*h* ratio across different temperature changes. The relevant geometric and material parameters are $L_{\alpha }=L_{\beta },$ $L_{\alpha }/h=10,$ $R_{\alpha }=R_{\beta },$ $\kappa_{p}=3,$ and $h=1\times 10^{-6}\;m$.

As shown in Figure 2, the lowest frequency increases with increasing the material length-scale parameter. This indicates that the material length-scale parameter stiffens the microscale shell, thereby raising its lowest frequency.

**Figure 3:**
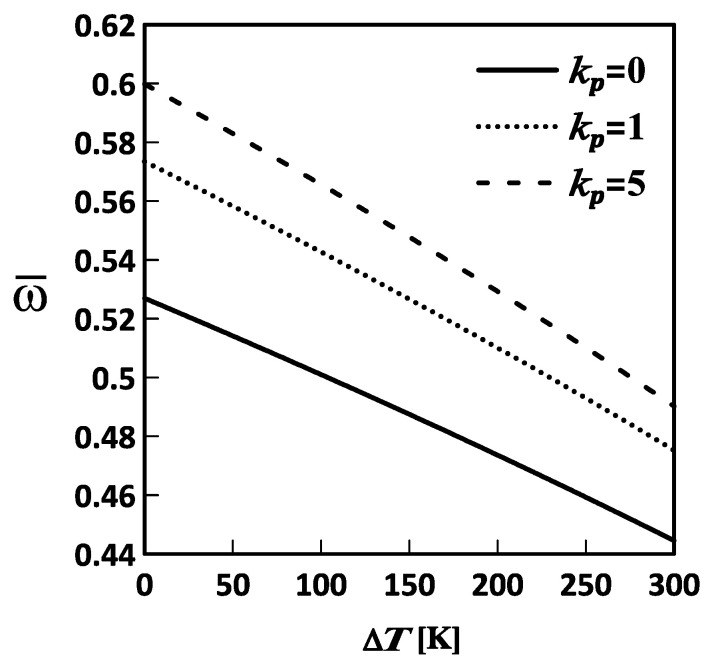
Variations in the lowest frequency of an FG-MEE DC microscale shell with the temperature change (ΔT) for the inhomogeneity index: $\kappa_{p}=$ 0, 1, and 5. DC: Doubly curved; FG: Functionally graded; MEE: Magneto-electro-elastic.

**Figure 4:**
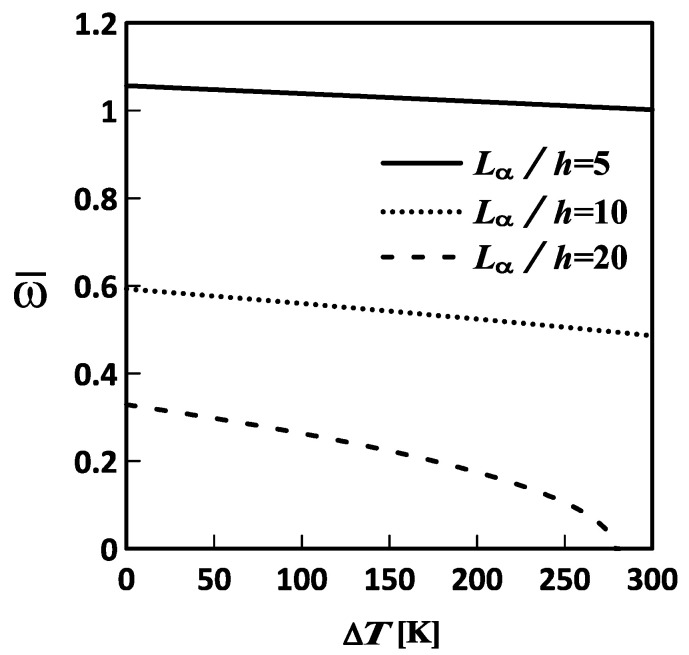
Variations in the lowest frequency of an FG-MEE DC microscale shell with the temperature change (ΔT) for the length-to-thickness ratio: $L_{\alpha }/h=$ 5, 10, and 20. DC: Doubly curved; FG: Functionally graded; MEE: Magneto-electro-elastic.

**Figure 5:**
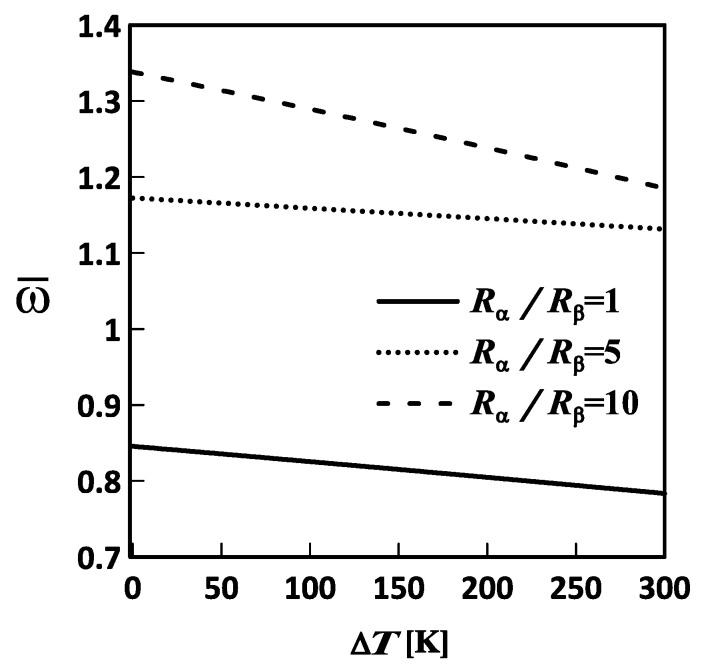
Variations in the lowest frequency of an FG-MEE DC microscale shell with the temperature change (ΔT) for the in-surface radius ratio: $R_{\alpha }/R_{\beta }=$ 1, 5, and 10. DC: Doubly curved; FG: Functionally graded; MEE: Magneto-electro-elastic.

Figure 3, Figure 4 and Figure 5 show the variations in the lowest frequency of the FG-MEE DC microscale shell with temperature changes for different inhomogeneity indices, length-to-thickness ratios, and mid-surface radius ratios, respectively. The relevant geometric and material parameters considered in Figure 3, Figure 4 and Figure 5 are $L_{\alpha }=L_{\beta },$ $L_{\alpha }/h=10,$ $R_{\alpha }=R_{\beta },$ l/h=0.5, $\kappa_{p}=0,\;1,\;\mathrm{and}\;5;$ $L_{\alpha }=L_{\beta },$ $L_{\alpha }/h=5,\;10,\;\mathrm{and}\;20,$ $R_{\alpha }=R_{\beta },$ l/h=0.5, $\kappa_{p}=3;$ and $L_{\alpha }=L_{\beta },$ $L_{\alpha }/h=10,$ $R_{\alpha }/R_{\beta }=1,\;5,\;\mathrm{and}\;10,$ l/h=0.5, $\kappa_{p}=3,$ respectively. Additionally, the plate thickness in these figures is set to $h=1\times 10^{-6}\;m$.

As shown in Figure 3, Figure 4 and Figure 5, the lowest frequency decreases with increasing temperature change. This indicates that an increase in temperature induces a set of in-surface compressive stresses in the microscale shell, softening it and thereby decreasing its lowest frequency. Additionally, the lowest frequency increases with increasing inhomogeneity index and mid-surface radius ratio, and decreasing length-to-thickness ratio. This indicated that an increase in the inhomogeneity index and the mid-surface radius ratio results in an increase in the volume fraction of the stiffer material, F, and in a deeper microscale shell, thereby increasing the overall stiffness of the microscale shell and, in turn, its lowest frequency. Moreover, an increase in the length-to-thickness ratio of the microscale shell makes it thinner, thereby reducing its overall stiffness and, in turn, decreasing its lowest frequency.

## 5. Conclusions

This work develops an EFG method based on the Galerkin weak formulation of the CCST to analyze the free vibration characteristics of an FG-MEE DC microscale shell subjected to a uniform temperature change. The current EFG method can be used to analyze the free-vibration characteristics of FG-MEE DC macroscale plates when the material length-scale parameter and the inverse of the mid-surface radius are set to zero.

In the comparison and validation study, the results show that the current EFG method converges rapidly and that its converged solutions closely agree with the exact 3D solutions for LC/FG-MEE macroscale plates reported in the literature. The results in the parametric study showed that the material length-scale parameter increases the overall stiffness of the FG-MEE DC microscale shell, thereby increasing its natural frequencies. The positive temperature change softens the FG-MEE DC microscale shell, thereby decreasing its natural frequency. Additionally, an increase in the inhomogeneity index increases the volume fraction of the stiffer material, F, thereby increasing the overall stiffness of the microscale shell and, in turn, its lowest frequency.

Future research can explore the thermal effects on other static and dynamic behaviors of the FG-MEE DC microscale shell, including thermal bending, buckling, postbuckling, and forced vibration. Additionally, investigating different boundary conditions on the thermally induced static and dynamic responses of the FG-MEE DC microscale shell is also recommended.

## Figures and Tables

**Table 1:** Elastic, piezoelectric, piezomagnetic, dielectric, and magnetic properties of piezoelectric and magnetostrictive materials.

Moduli	BaTiO_3_ [50,51]	CoFe_2_O_4_ [50,51]
$c_{11}=c_{22}$ [Pa]	$166.0\times 10^{9}$	$286.0\times 10^{9}$
$c_{33}$ [Pa]	$162.0\times 10^{9}$	$269.5\times 10^{9}$
$c_{12}$ [Pa]	$77.0\times 10^{9}$	$173.0\times 10^{9}$
$c_{13}=c_{23}$ [Pa]	$78.0\times 10^{9}$	$170.5\times 10^{9}$
$c_{44}=c_{55}$ [Pa]	$43.0\times 10^{9}$	$45.3\times 10^{9}$
$c_{66}$ [Pa]	$44.5\times 10^{9}$	$56.5\times 10^{9}$
$e_{31}=e_{32}$ $\left[Cm^{-2}\right]$	−4.4	0.0
$e_{33}$ $\left[Cm^{-2}\right]$	18.6	0.0
$e_{24}=e_{15}$ $\left[Cm^{-2}\right]$	11.6	0.0
$q_{31}=q_{32}$ $\left[NA^{-1}m^{-1}\right]$	0.0	580.3
$q_{33}$ $\left[NA^{-1}m^{-1}\right]$	0.0	699.7
$q_{24}=q_{15}$ $\left[N{\left(\mathrm{Am}\right)}^{-1}\right]$	0.0	550.0
$\eta_{11}=\eta_{22}$ $\left[C^{2}N^{-1}m^{-2}\right]$	$11.2\times 10^{-9}$	$0.08\times 10^{-9}$
$\eta_{33}$ $\left[C^{2}N^{-1}m^{-2}\right]$	$12.6\times 10^{-9}$	$0.093\times 10^{-9}$
$\beta_{11}=\beta_{22}$ $\left[Ns^{2}C^{-2}\right]$	$5.0\times 10^{-6}$	$-590.0\times 10^{-6}$
$\beta_{33}$ $\left[Ns^{2}C^{-2}\right]$	$10.0\times 10^{-6}$	$157.0\times 10^{-6}$
$d_{11}=d_{22}=d_{33}\;$ $\left[NsC^{-1}V^{-1}\right]$	0.0	0.0
$\alpha_{1}=\alpha_{2}$ $\left[K^{-1}\right]$	$15.7\times 10^{-6}$	$10.0\times 10^{-6}$
$\alpha_{3}$ $\left[K^{-1}\right]$	$6.4\times 10^{-6}$	$10.0\times 10^{-6}$
ρ $\left[\mathrm{kg}m^{-3}\right]$	5800.0	5300.0
*l* [m]	$17.6\times 10^{-6}$	$17.6\times 10^{-6}$

**Table 2:** The first five frequency parameters ${\bar{\omega }}_{i}\;\left(i=1-5\right)$ of sandwich piezoelectric and magnetostrictive macro- and microscale plates.

Laminates	Surface Conditions	*l*/*h*	Theories	${\bar{\omega }}_{1}$	${\bar{\omega }}_{2}$	${\bar{\omega }}_{3}$	${\bar{\omega }}_{4}$	${\bar{\omega }}_{5}$
B/F/B	Case 1	0	Current EFG method with $n_{p}$ = 21	0.9578 (0.30%)	1.8563 (0.04%)	3.2296 (0.05%)	4.4454 (1.15%)	5.1701 (0.86%)
		0	Current EFG method with $n_{p}$ = 31	0.9587 (0.19%)	1.8537 (0.10%)	3.2254 (0.08%)	4.4718 (0.56%)	5.1909 (0.46%)
		0	Modified Pagane method [52]	0.9606	1.8556	3.2279	4.4972	5.2151
		0.25	Current EFG method with $n_{p}$ = 31	1.0909	1.8696	3.236	4.6848	5.4321
		0.5	Current EFG method with $n_{p}$ = 31	1.3857	1.8955	3.2495	4.8418	5.701
		1	Current EFG method with $n_{p}$ = 31	1.9275	2.0513	3.2632	4.9362	6.317
B/F/B	Case 2	0	Current EFG method with $n_{p}$ = 21	0.9624 (0.29%)	1.8563 (0.04%)	3.2371 (0.06%)	4.4454 (1.15%)	5.3262 (0.97%)
		0	Current EFG method with $n_{p}$ = 31	0.9637 (0.15%)	1.8537 (0.10%)	3.2332 (0.07%)	4.4718 (0.56%)	5.3612 (0.32%)
		0	State space method [50]	0.9652	1.8556	3.2353	4.4972	5.3786
		0	Modified Pagane method [52]	0.9652	1.8556	3.2353	4.4972	5.3786
		0.25	Current EFG method with $n_{p}$ = 31	1.099	1.8696	3.2464	4.6848	5.674
		0.5	Current EFG method with $n_{p}$ = 31	1.4022	1.8955	3.2661	4.8418	6.0219
		1	Current EFG method with $n_{p}$ = 31	1.9275	2.103	3.2885	4.9362	6.6533
F/B/F	Case 1	0	Current EFG method with $n_{p}$ = 21	1.0616 (0.46%)	1.9589 (0.04%)	3.3866 (0.04%)	4.6945 (1.01%)	5.8315 (1.07%)
		0	Current EFG method with $n_{p}$ = 31	1.0636 (0.27%)	1.9618 (0.10%)	3.3904 (0.08%)	4.7270 (0.33%)	5.8624 (0.55%)
		0	Modified Pagane method [52]	1.0665	1.9598	3.3878	4.7424	5.8946
		0.25	Current EFG method with $n_{p}$ = 31	1.1949	1.9798	3.4116	4.9651	6.1269
		0.5	Current EFG method with $n_{p}$ = 31	1.4894	2.0121	3.4449	5.1559	6.4094
		1	Current EFG method with $n_{p}$ = 31	2.054	2.1751	3.4807	5.2715	7.0609
F/B/F	Case 2	0	Current EFG method with $n_{p}$ = 21	1.0623 (0.46%)	1.9589 (0.04%)	3.3868 (0.03%)	4.6945 (1.01%)	5.8362 (1.06%)
		0	Current EFG method with $n_{p}$ = 31	1.0642 (0.28%)	1.9618 (0.10%)	3.3906 (0.08%)	4.7270 (0.33%)	5.8643 (0.59%)
		0	State space method [50]	1.0672	1.9598	3.3879	4.7424	5.8990
		0	Modified Pagane method [52]	1.0672	1.9598	3.3879	4.7424	5.8990
		0.25	Current EFG method with $n_{p}$ = 31	1.1954	1.9798	3.4117	4.9651	6.1255
		0.5	Current EFG method with $n_{p}$ = 31	1.4894	2.0121	3.4447	5.1559	6.401
		1	Current EFG method with $n_{p}$ = 31	2.054	2.1737	3.4799	5.2715	7.0492

The numbers in parentheses are the relative errors between the current solutions and those obtained with the modified Pagano method [52]. B: BaTiO_3_ material; EFG: Element-free Galerkin; F: CoFe_2_O_4_ material.

**Table 3:** The first five frequency parameters ${\bar{\omega }}_{i}\;(i=1-5)$ of FG-MEE rectangular plates with surface conditions of Case 2.

$2h/L_{\alpha }$	$\kappa_{p}$	*l*/*h*	Theories	${\bar{\omega }}_{1}$	${\bar{\omega }}_{2}$	${\bar{\omega }}_{3}$	${\bar{\omega }}_{4}$	${\bar{\omega }}_{5}$
0.1	1	0	Current EFG method with $n_{p}$ = 21	9.5389 (0.16%)	28.8388 (0.00%)	51.2705 (0.00%)	122.4957 (1.25%)	135.9125 (1.28%)
		0	Current EFG method with $n_{p}$ = 31	9.5466 (0.08%)	28.8389 (0.00%)	51.2711 (0.00%)	123.4124 (0.51%)	136.9754 (0.50%)
		0	Modified Pagano solutions [52].	9.5543	28.8389	51.2717	124.0428	137.6705
		0	Discrete layer solutions [51]	9.525	28.762	50.966	131.186	139.106
		0.25	Current EFG method with $n_{p}$ = 31	10.6637	28.91	51.3336	129.01	143.8911
		0.5	Current EFG method with $n_{p}$ = 31	13.3594	29.0352	51.439	133.3179	150.2874
		1	Current EFG method with $n_{p}$ = 31	20.3639	29.2001	51.5722	135.9741	158.8259
0.1	3	0	Current EFG method with $n_{p}$ = 21	9.7569 (0.16%)	30.0245 (0.00%)	53.1627 (0.00%)	124.5930 (1.24%)	134.2890 (0.94%)
		0	Current EFG method with $n_{p}$ = 31	9.7650 (0.08%)	30.0245 (0.00%)	53.1632 (0.00%)	125.5131 (0.51%)	135.2110 (0.26%)
		0	Modified Pagano solutions [52].	9.7730	30.0246	53.1637	126.1601	135.8650
		0	Discrete layer solutions [51]	9.747	29.975	53.008	128.667	136.634
		0.25	Current EFG method with $n_{p}$ = 31	10.9368	30.0934	53.228	131.075	141.9602
		0.5	Current EFG method with $n_{p}$ = 31	13.7465	30.2131	53.3368	135.293	148.3608
		1	Current EFG method with $n_{p}$ = 31	20.9663	30.3699	53.4761	137.8808	157.3409
0.2	1	0	Current EFG method with $n_{p}$ = 21	7.9228 (0.41%)	14.4082 (0.00%)	25.0166 (0.02%)	33.0838 (1.07%)	40.7352 (0.96%)
		0	Current EFG method with $n_{p}$ = 31	7.9381 (0.21%)	14.4084 (0.00%)	25.0189 (0.01%)	33.2970 (0.44%)	40.9764 (0.37%)
		0	Modifies Pagano solutions [52].	7.9552	14.4086	25.0208	33.4432	41.1283
		0	Discrete layer solutions [51]	7.942	14.371	24.968	35.106	41.506
		0.25	Current EFG method with $n_{p}$ = 31	8.9886	14.5497	25.1917	34.9376	43.1596
		0.5	Current EFG method with $n_{p}$ = 31	11.3324	14.791	25.4515	36.2081	45.5394
		1	Current EFG method with $n_{p}$ = 31	15.094	16.7019	25.7218	36.9787	50.4366
0.2	3	0	Current EFG method with $n_{p}$ = 21	8.0174 (0.40%)	15.0027 (0.00%)	25.8847 (0.02%)	33.7028 (1.06%)	40.6723 (0.82%)
		0	Current EFG method with $n_{p}$ = 31	8.0326 (0.21%)	15.0028 (0.00%)	25.8868 (0.01%)	33.9151 (0.44%)	40.8711 (0.34%)
		0	Modified Pagano solutions [52].	8.0497	15.0030	25.8888	34.0653	41.0095
		0	Discrete layer solutions [51]	8.037	14.978	25.851	34.648	41.218
		0.25	Current EFG method with $n_{p}$ = 31	9.134	15.1395	26.0647	35.5384	43.024
		0.5	Current EFG method with $n_{p}$ = 31	11.5655	15.3701	26.3352	36.7766	45.4627
		1	Current EFG method with $n_{p}$ = 31	15.6578	17.0115	26.6213	37.5238	50.7366

The numbers in parentheses are the relative errors between the current solutions and those obtained with the modified Pagano method [52]. EFG: Element-free Galerkin; FG: Functionally graded; MEE: Magneto-electro-elastic.

## Data Availability

The original contributions presented in this study are included in the article. Further inquiries can be directed to the corresponding author.

## Abbreviations/Notations

The following abbreviations and notations are used in this paper:

B	BaTiO_3_ material
CCM	Classical continuum mechanics
CCST	Consistent couple stress theory
DC	Doubly curved
DRK	Differential reproducing kernel
EFG	Element-free Galerkin
F	CoFe_2_O_4_ material
FEM	Finite element method
FG	Functionally graded
FSDT	First-order shear deformation theory
LC	Laminated composite
MCST	Modified couple stress theory
MEE	Magneto-electro-elastic
MEMS	Micro-electro-mechanical system
RSDT	Refined shear deformation theory
SSDT	Sinusoidal shear deformation theory
2D	Two-dimensional
3D	Three-dimensional
Notations	
$a_{l}$	The size of the influence zone for the reference point *l*
*A*	The DC microshell’s in-surface domain
$b_{kk}$	The coupling couple stress and skew-symmetric parts of the curvature coefficients
$b_{0}\left(\xi \right)$	Undetermined function vector
$B_{k}$	Magnetic flux tensor
$B_{i}$	Relevant matrices relating dependent variables and primary variables
$c_{ij}$	The elastic coefficients
$c_{\alpha k}$	The coupling force-stress-temperature change coefficients
$d_{kk}$	The piezomagnetic coefficients
$D_{k}$	Electric flux tensor
$D_{p}$	In-surface electric flux vector
$e_{ij}$	The piezoelectric coefficients
$e_{\alpha 3}$	The coupling elastic flux and temperature change coefficients
$E_{k}$	Electric field tensor
$E_{p}$	In-surface electric field vector
$f^{a}\left(\xi \right)$	DRK interpolant
$g_{ij}$	Geometric stiffness matrix coefficients
$G_{ij}$	Shear modulus of the *ij*-surface
*h*	DC microshell’s thickness
$H_{k}$	Magnetic field tensor
$H_{p}$	In-surface magnetic field vector
$I_{R}$	DC microshell’s total potential energy
$k_{ij}$	Stiffness matrix coefficients
$L_{\alpha },\;L_{\beta }$	DC microshell’s in-surface dimensions
*m*	Layer number
$\hat{m},\;\hat{n}$	Wave numbers in the α and β directions, respectively
$m_{kk}$	Mass matrix coefficients
$n_{p}$	Number of sampling points
$N_{l}\left(\xi \right)$	Shape function corresponding to the reference point *l*
$P\left(\xi -\xi_{l}\right)$	Base function set
$q_{ij}$	The magnetoelastic coefficients
$q_{\alpha 3}$	The coupling magnetic flux and temperature change coefficients
$Q_{i}$	Relevant matrices relating the generalized stresses and strains
$R_{\alpha },\;R_{\beta }$	DC microshell’s radii in α and β directions, respectively
*t*	The time variable
**u**	The in-surface displacement vector
$u_{\alpha },\;u_{\beta },\;u_{\gamma }$	Displacement components in the α, β, andγ directions, respectively
$U_{s}$	DC microshell’s strain energy
*V*	DC microshell’s kinetic energy
$w_{a}\left(\xi -\xi_{l}\right)$	Gaussian function
*W*	Work done due to the thermally induced initial stresses
α, β, γ	The doubly curved shell coordinates
$\beta_{kk}$	The magnetic permeability coefficients
$\gamma_{\alpha },\;\gamma_{\beta }$	Scale factors for the DC shell coordinate system
$\gamma_{ij}$	Shear strain tensor
$\Gamma_{B},\;\Gamma_{F}$	Volume fractions of BaTiO_3_ and CoFe_2_O_4_
δ	The variational operator
ΔT	Temperature change
$\varepsilon_{ij}$	Normal strain tensor
$\varepsilon_{kk}^{nl}$	Full nonlinear strain tensor
$\varepsilon_{p}$	In-surface strain vector
$\varepsilon_{s}$	Tranverse shear strain vector
ζ	Thickness coordinate
$\eta_{kk}$	The dielectric permeability coefficients
$\theta_{k}$	Rotation tensor
$\kappa_{k}$	Skew-symmetric parts of the curvature tensor
$\kappa_{p}$	The inhomogeneity index
κ	Skew-symmetric curvature vector
$\mu_{k}$	Couple stress tensor
μ	Couple stress vector
ξ	Natural coordinate
ρ	Mass density
$\sigma_{\left(ij\right)}$	Symmetric parts of the force-stress tensor
$\sigma_{\left[ij\right]}$	Skew-symmetric parts of the force-stress tensor
$\sigma_{p}$	The in-surface force-stress tensor
$\sigma_{s}$	Transverse force-stress vector
$\phi_{l}\left(\xi \right)$	Enrichment function corresponding to the reference point *l*
Φ	The electric potential
$\psi_{l}\left(\xi \right)$	Primitive function corresponding to the reference point *l*
Ψ	The magnetic potential
ω	Natural frequency
$\bar{\omega }$	Dimensionless natural frequency

## Appendix A. Relevant Matrices and Vectors Shown in Equations (33)–(47)

Relevant matrices and vectors shown in Equations (33)–(47) are expressed as follows:

(A1) $$B_{p}={\left\{\begin{array}{cc} B_{\alpha } & B_{\beta } \end{array}\right\}}^{T}=Q_{q}\varepsilon_{s}+Q_{d}E_{p}+Q_{\beta }H_{p},$$

(A2) $$B_{\zeta }={\left(Q_{2}\right)}^{T}\varepsilon_{p}+q_{33}\varepsilon_{\zeta \zeta }+d_{33}E_{\zeta }+\beta_{33}H_{\zeta }-q_{\alpha 3}\Delta T,$$

(A3) $$Q_{p}=\left[\begin{array}{ccc} c_{11} & c_{12} & 0\\ c_{12} & c_{22} & 0\\ 0 & 0 & c_{66} \end{array}\right],$$

(A4) $$Q_{\zeta }={\left\{\begin{array}{ccc} c_{13} & c_{23} & 0 \end{array}\right\}}^{T},$$

(A5) $$Q_{s}=\left[\begin{array}{cc} c_{44} & 0\\ 0 & c_{55} \end{array}\right],$$

(A6) $$Q_{c}=\left[\begin{array}{ccc} 2G_{32}\;l^{2} & 0 & 0\\ 0 & 2G_{13}\;l^{2} & 0\\ 0 & 0 & 2G_{21}\;l^{2} \end{array}\right],$$

(A7) $$Q_{d}=\left[\begin{array}{cc} d_{11} & 0\\ 0 & d_{22} \end{array}\right],$$

(A8) $$Q_{e}=\left[\begin{array}{cc} 0 & e_{24}\\ e_{15} & 0 \end{array}\right],$$

(A9) $$Q_{q}=\left[\begin{array}{cc} 0 & q_{24}\\ q_{15} & 0 \end{array}\right],$$

(A10) $$Q_{\eta }=\left[\begin{array}{cc} \eta_{11} & 0\\ 0 & \eta_{22}, \end{array}\right],$$

(A11) $$Q_{\beta }=\left[\begin{array}{cc} \beta_{11} & 0\\ 0 & \beta_{22}, \end{array}\right],$$

(A12) $$B_{1}=\left[\begin{array}{cc} \gamma_{\alpha }^{-1}\partial_{\alpha } & 0\\ 0 & \gamma_{\beta }^{-1}\partial_{\beta }\\ \gamma_{\beta }^{-1}\partial_{\beta } & \gamma_{\alpha }^{-1}\partial_{\alpha } \end{array}\right],$$

(A13) $$B_{2}={\left\{\begin{array}{ccc} {\left(R_{\alpha }\gamma_{\alpha }\right)}^{-1} & {\left(R_{\beta }\gamma_{\beta }\right)}^{-1} & 0 \end{array}\right\}}^{T},$$

(A14) $$B_{3}=\left[\begin{array}{cc} \partial_{\zeta }-{\left(R_{\alpha }\gamma_{\alpha }\right)}^{-1} & 0\\ 0 & \partial_{\zeta }-{\left(R_{\beta }\gamma_{\beta }\right)}^{-1} \end{array}\right],$$

(A15) $$B_{4}={\left\{\begin{array}{cc} \gamma_{\alpha }^{-1}\partial_{\alpha } & \gamma_{\beta }^{-1}\partial_{\beta } \end{array}\right\}}^{T},$$

(A16) $$B_{5}=\left\{\partial_{\zeta }\right\},$$

(A17) $$B_{6}=\left[\begin{array}{cc} b_{11} & b_{12}\\ b_{21} & b_{22}\\ b_{31} & b_{32} \end{array}\right],$$

(A18) $$B_{7}={\left\{\begin{array}{ccc} d_{11} & d_{21} & d_{31} \end{array}\right\}}^{T},$$

(A19) $$B_{8}={\left\{\begin{array}{cc} -\gamma_{\alpha }^{-1}\partial_{\alpha } & -\gamma_{\beta }^{-1}\partial_{\beta } \end{array}\right\}}^{T},$$

(A20) $$\begin{array}{ll} b_{11}= & -{\left(4\gamma_{\beta }^{2}\right)}^{-1}\partial_{\beta \beta }-\left(1/4\right)\partial_{\zeta \zeta }-\left[{\left(4R_{\alpha }\gamma_{\alpha }\right)}^{-1}+{\left(4R_{\beta }\gamma_{\beta }\right)}^{-1}\right]\partial_{\zeta }\\ & +\left[{\left(4R_{\alpha }^{2}\gamma_{\alpha }^{2}\right)}^{-1}-{\left(4R_{\alpha }R_{\beta }\gamma_{\alpha }\gamma_{\beta }\right)}^{-1}\right], \end{array}$$

(A21) $$b_{12}=b_{21}={\left(4\gamma_{\alpha }\gamma_{\beta }\right)}^{-1}\partial_{\alpha \beta },$$

(A22) $$\begin{array}{ll} b_{22}= & -{\left(4\gamma_{\alpha }^{2}\right)}^{-1}\partial_{\alpha \alpha }-\left(1/4\right)\partial_{\zeta \zeta }-\left[{\left(4R_{\alpha }\gamma_{\alpha }\right)}^{-1}+{\left(4R_{\beta }\gamma_{\beta }\right)}^{-1}\right]\partial_{\zeta }\\ & +\left[{\left(4R_{\beta }^{2}\gamma_{\beta }^{2}\right)}^{-1}-{\left(4R_{\alpha }R_{\beta }\gamma_{\alpha }\gamma_{\beta }\right)}^{-1}\right], \end{array}$$

(A23) $$b_{31}=\left[{\left(4R_{\alpha }\gamma_{\alpha }^{2}\right)}^{-1}\partial_{\alpha }+{\left(4\gamma_{\alpha }\right)}^{-1}\partial_{\alpha \zeta }\right],$$

(A24) $$b_{32}=\left[{\left(4R_{\beta }\gamma_{\beta }^{2}\right)}^{-1}\partial_{\beta }+{\left(4\gamma_{\beta }\right)}^{-1}\partial_{\beta \zeta }\right],$$

(A25) $$d_{11}=\left[-{\left(4R_{\alpha }\gamma_{\alpha }^{2}\right)}^{-1}+{\left(4R_{\beta }\gamma_{\alpha }\gamma_{\beta }\right)}^{-1}\right]\partial_{\alpha }+\left[{\left(4\gamma_{\alpha }\right)}^{-1}\partial_{\alpha \zeta }\right],$$

(A26) $$d_{21}=\left[-{\left(4R_{\beta }\gamma_{\beta }^{2}\right)}^{-1}+{\left(4R_{\alpha }\gamma_{\alpha }\gamma_{\beta }\right)}^{-1}\right]\partial_{\beta }+\left[{\left(4\gamma_{\beta }\right)}^{-1}\partial_{\beta \zeta }\right],$$

(A27) $$d_{31}=-{\left(4\gamma_{\alpha }^{2}\right)}^{-1}\partial_{\alpha \alpha }-{\left(4\gamma_{\beta }^{2}\right)}^{-1}\partial_{\beta \beta }.$$

## Appendix B. Detailed Expressions of *k_ij_*, *m_kk_*, and *g_ij_*

Detailed expressions of *k_ij_*, *m_kk_*, and *g_ij_* are provided as follows:

(A28) k11=∫−h/2h/2m˜2c11NiNj/γα2+n˜2c66NiNj/γβ2+c55Ni,ζ−Ni/RαγαNj,ζ−Nj/Rαγαγαγβdζ +l2/4∫−h/2h/2G32n˜2Ni/γβ2−Ni,ζζ−Ni,ζ/Rαγα−Ni,ζ/Rβγβ+Ni/Rα2γα2−Ni/RαγαRβγβ n˜2Nj/γβ2−Nj,ζζ−Nj,ζ/Rαγα−Nj,ζ/Rβγβ+Nj/Rα2γα2−Nj/RαγαRβγβ +G13m˜2n˜2NiNj/γα2γβ2+G21m˜Ni/Rαγα2+m˜Ni,ζ/γαm˜Nj/Rαγα2+m˜Nj,ζ/γαγαγβdζ,

(A29) $$\begin{array}{ll} k_{12} & =\int_{-h/2}^{h/2}\left\{\tilde{m}\tilde{n}\left(c_{12}+c_{66}\right)N_{i}N_{j}\right\}d\zeta \\ & +\left(l^{2}/4\right)\int_{-h/2}^{h/2}\left\{G_{32}\right.\left[{\tilde{n}}^{2}N_{i}/\gamma_{\beta }^{2}-N_{i,\zeta \zeta }-N_{i,\zeta }/\left(R_{\alpha }\gamma_{\alpha }\right)-N_{i,\zeta }/\left(R_{\beta }\gamma_{\beta }\right)+N_{i}/\left(R_{\alpha }^{2}\gamma_{\alpha }^{2}\right)-N_{i}/\left(R_{\alpha }\gamma_{\alpha }R_{\beta }\gamma_{\beta }\right)\right]\\ & \left[-\tilde{m}\tilde{n}N_{j}/\left(\gamma_{\alpha }\gamma_{\beta }\right)\right]-G_{13}\left[\tilde{m}\tilde{n}N_{i}/\left(\gamma_{\alpha }\gamma_{\beta }\right)\right]\left[{\tilde{m}}^{2}N_{j}/\gamma_{\alpha }^{2}-N_{j,\zeta \zeta }-N_{j,\zeta }/\left(R_{\alpha }\gamma_{\alpha }\right)-N_{j,\zeta }/\left(R_{\beta }\gamma_{\beta }\right)+N_{j}/\left(R_{\alpha }^{2}\gamma_{\alpha }^{2}\right)\right.\\ & \left.\left.-N_{j}/\left(R_{\alpha }\gamma_{\alpha }R_{\beta }\gamma_{\beta }\right)\right]+G_{21}\left[\left(\tilde{m}N_{i}\right)/\left(R_{\alpha }\gamma_{\alpha }^{2}\right)+\left(\tilde{m}N_{i,\zeta }\right)/\gamma_{\alpha }\right]\left[\left(\tilde{n}N_{j}\right)/\left(R_{\beta }\gamma_{\beta }^{2}\right)+\left(\tilde{n}N_{j,\zeta }\right)/\gamma_{\beta }\right]\right\}\gamma_{\alpha }\gamma_{\beta }d\zeta , \end{array}$$

(A30) $$\begin{array}{ll} k_{13} & =\int_{-h/2}^{h/2}\left\{\left(-\tilde{m}N_{i}/\gamma_{\alpha }\right)\left[c_{11}N_{j}/\left(R_{\alpha }\gamma_{\alpha }\right)+c_{12}N_{j}/\left(R_{\beta }\gamma_{\beta }\right)+c_{13}N_{j,\zeta }\right]\gamma_{\alpha }\gamma_{\beta }+\left[N_{i,\zeta }-N_{i}/\left(R_{\alpha }\gamma_{\alpha }\right)\right]\left(\tilde{m}c_{55}\gamma_{\beta }N_{j}\right)\right\}d\zeta \\ & +\left(l^{2}/4\right)\int_{-h/2}^{h/2}\left\{G_{32}\right.\left[{\tilde{n}}^{2}N_{i}/\gamma_{\beta }^{2}-N_{i,\zeta \zeta }-N_{i,\zeta }/\left(R_{\alpha }\gamma_{\alpha }\right)-N_{i,\zeta }/\left(R_{\beta }\gamma_{\beta }\right)+N_{i}/\left(R_{\alpha }^{2}\gamma_{\alpha }^{2}\right)-N_{i}/\left(R_{\alpha }\gamma_{\alpha }R_{\beta }\gamma_{\beta }\right)\right]\\ & \left[-\tilde{m}N_{j}/\left(R_{\alpha }\gamma_{\alpha }^{2}\right)+\tilde{m}N_{j}/\left(R_{\beta }\gamma_{\alpha }\gamma_{\beta }\right)+\tilde{m}N_{j,\zeta }/\gamma_{\alpha }\right]+G_{13}\left[-\tilde{m}\tilde{n}N_{i}/\left(\gamma_{\alpha }\gamma_{\beta }\right)\right]\left[\left(-\tilde{n}N_{j}\right)/\left(R_{\beta }\gamma_{\beta }^{2}\right)+\left(\tilde{n}N_{j}\right)/\left(R_{\alpha }\gamma_{\alpha }\gamma_{\beta }\right)\right.\\ & \left.\left.+\left(\tilde{n}N_{j,\zeta }\right)/\gamma_{\beta }\right]+G_{21}\left[-\tilde{m}N_{i}/\left(R_{\alpha }\gamma_{\alpha }^{2}\right)-\tilde{m}N_{i,\zeta }/\gamma_{\alpha }\right]\left[{\tilde{m}}^{2}N_{j}/\gamma_{\alpha }^{2}+{\tilde{n}}^{2}N_{j}/\gamma_{\beta }^{2}\right]\right\}\gamma_{\alpha }\gamma_{\beta }d\zeta , \end{array}$$

(A31) $$k_{14}=\int_{-h/2}^{h/2}\left\{-\tilde{m}\gamma_{\beta }e_{31}N_{i}N_{j,\zeta }+\tilde{m}\gamma_{\beta }e_{15}\left[N_{i,\zeta }-N_{i}/\left(R_{\alpha }\gamma_{\alpha }\right)\right]N_{j}\right\}d\zeta ,$$

(A32) $$k_{15}=\int_{-h/2}^{h/2}\left\{-\tilde{m}\gamma_{\beta }q_{31}N_{i}N_{j,\zeta }+\tilde{m}\gamma_{\beta }q_{15}\left[N_{i,\zeta }-N_{i}/\left(R_{\alpha }\gamma_{\alpha }\right)\right]N_{j}\right\}d\zeta ,$$

(A33) $$k_{21}\left(N_{i},\;N_{j}\right)=k_{12}\left(N_{j},N_{i}\right),$$

(A34) k22=∫−h/2h/2n˜2c22NiNj/γβ2+m˜2c66NiNj/γα2+c44Ni,ζ−Ni/RβγβNj,ζ−Nj/Rβγβγαγβdζ +l2/4∫−h/2h/2G32m˜2n˜2NiNj/γβ2γβ2+G13m˜2Ni/γα2−Ni,ζζ−Ni,ζ/Rαγα−Ni,ζ/Rβγβ +Ni/Rβ2γβ2−Ni/RαγαRβγβm˜2Nj/γα2−Nj,ζζ−Nj,ζ/Rαγα−Nj,ζ/Rβγβ +Nj/Rβ2γβ2−Nj/RαγαRβγβ+G21n˜Ni/Rβγβ2+n˜Ni,ζ/γβ n˜Nj/Rβγβ2+n˜Nj,ζ/γβγαγβdζ,

(A35) k23=∫−h/2h/2−n˜Ni/γβc12Nj/Rαγα+c22Nj/Rβγβ+c23Nj,ζγαγβ+Ni,ζ−Ni/Rβγβn˜c44γαNjdζ +l2/4∫−h/2h/2G32−m˜n˜Ni/γαγβ−m˜Nj/Rαγα2+m˜Nj/Rαγαγβ+m˜Nj,ζ/γα +G13m˜2Ni/γα2−Ni,ζζ−Ni,ζ/Rαγα−Ni,ζ/Rβγβ+Ni/Rβ2γβ2−Ni/RαγαRβγβ −n˜Nj/Rβγβ2+n˜Nj/Rβγαγβ+n˜Nj,ζ/γβ+G21−n˜Ni/Rβγβ2−n˜Nj,ζ/γβ m˜2Nj/γα2+n˜2Nj/γβ2γαγβdζ,

(A36) $$k_{24}=\int_{-h/2}^{h/2}\left\{-\tilde{n}\gamma_{\alpha }e_{32}N_{i}N_{j,\zeta }+\tilde{n}\gamma_{\alpha }e_{24}\left[N_{i,\zeta }-N_{i}/\left(R_{\beta }\gamma_{\beta }\right)\right]N_{j}\right\}d\zeta ,$$

(A37) $$k_{25}=\int_{-h/2}^{h/2}\left\{-\tilde{n}\gamma_{\alpha }q_{32}N_{i}N_{j,\zeta }+\tilde{n}\gamma_{\alpha }q_{24}\left[N_{i,\zeta }-N_{i}/\left(R_{\beta }\gamma_{\beta }\right)\right]N_{j}\right\}d\zeta ,$$

(A38) $$k_{31}\left(N_{i},\;N_{j}\right)=k_{13}\left(N_{j},\;N_{i}\right),$$

(A39) $$k_{32}\left(N_{i},\;N_{j}\right)=k_{23}\left(N_{j},\;N_{i}\right),$$

(A40) $$\begin{array}{ll} k_{33} & =\int_{-h/2}^{h/2}\left\{\left[N_{i}/\left(R_{\alpha }\gamma_{\alpha }\right)\right]\left[c_{11}N_{j}/\left(R_{\alpha }\gamma_{\alpha }\right)+c_{12}N_{j}/\left(R_{\beta }\gamma_{\beta }\right)+c_{13}N_{j,\zeta }\right]+\left[N_{i}/\left(R_{\beta }\gamma_{\beta }\right)\right]\left[c_{12}N_{j}/\left(R_{\alpha }\gamma_{\alpha }\right)\right.\right.\\ & +c_{22}N_{j}/\left(R_{\beta }\gamma_{\beta }\right)+\left.c_{23}N_{j,\zeta }\right]+\left(\tilde{m}N_{i}/\gamma_{\alpha }\right)\left(\tilde{m}c_{55}N_{j}/\gamma_{\alpha }\right)+\left(\tilde{n}N_{i}/\gamma_{\beta }\right)\left(\tilde{n}c_{44}N_{j}/\gamma_{\beta }\right)+N_{i,\zeta }\left[c_{13}N_{j}/\left(R_{\alpha }\gamma_{\alpha }\right)\right.\\ & \left.\left.+c_{23}N_{j}/\left(R_{\beta }\gamma_{\beta }\right)+c_{33}N_{j,\zeta }\right]\right\}\gamma_{\alpha }\gamma_{\beta }d\zeta \\ & +\left(l^{2}/4\right)\int_{-h/2}^{h/2}\left\{G_{32}\right.\left[-\tilde{m}N_{i}/\left(R_{\alpha }\gamma_{\alpha }^{2}\right)+\tilde{m}N_{i}/\left(R_{\beta }\gamma_{\alpha }\gamma_{\beta }\right)+\tilde{m}N_{i,\zeta }/\gamma_{\alpha }\right]\left[-\tilde{m}N_{j}/\left(R_{\alpha }\gamma_{\alpha }^{2}\right)+\tilde{m}N_{j}/\left(R_{\beta }\gamma_{\alpha }\gamma_{\beta }\right)\right.\\ & \left.+\tilde{m}N_{j,\zeta }/\gamma_{\alpha }\right]+G_{13}\left[-\tilde{n}N_{i}/\left(R_{\beta }\gamma_{\beta }^{2}\right)+\tilde{n}N_{i}/\left(R_{\alpha }\gamma_{\alpha }\gamma_{\beta }\right)+\tilde{n}N_{i,\zeta }/\gamma_{\beta }\right]\left[-\tilde{n}N_{j}/\left(R_{\beta }\gamma_{\beta }^{2}\right)+\tilde{n}N_{j}/\left(R_{\alpha }\gamma_{\alpha }\gamma_{\beta }\right)\right.\\ & \left.+\left.\tilde{n}N_{j,\zeta }/\gamma_{\beta }\right]+G_{21}\left[\left({\tilde{m}}^{2}N_{i}\right)/\gamma_{\alpha }^{2}+\left({\tilde{n}}^{2}N_{i}\right)/\gamma_{\beta }^{2}\right]\left[\left({\tilde{m}}^{2}N_{j}\right)/\gamma_{\alpha }^{2}+\left({\tilde{n}}^{2}N_{j}\right)/\gamma_{\beta }^{2}\right]\right\}\gamma_{\alpha }\gamma_{\beta }d\zeta , \end{array}$$

(A41) $$\begin{array}{ll} k_{34} & =\int_{-h/2}^{h/2}\left\{\left[\gamma_{\beta }e_{31}N_{i}/R_{\alpha }+\gamma_{\alpha }e_{32}N_{i}/R_{\beta }\right]N_{j,\zeta }+\left[{\tilde{m}}^{2}\right.\right.\gamma_{\beta }e_{15}N_{i}/\gamma_{\alpha }\\ & \left.\left.+{\tilde{n}}^{2}\gamma_{\alpha }e_{24}N_{i}/\gamma_{\beta }\right]N_{j}+e_{33}\gamma_{\alpha }\gamma_{\beta }N_{i,\zeta }N_{j,\zeta }\right\}d\zeta , \end{array}$$

(A42) $$\begin{array}{ll} k_{35} & =\int_{-h/2}^{h/2}\left\{\left[\gamma_{\beta }q_{31}N_{i}/R_{\alpha }+\gamma_{\alpha }q_{32}N_{i}/R_{\beta }\right]N_{j,\zeta }+\left[{\tilde{m}}^{2}\right.\right.\gamma_{\beta }q_{15}N_{i}/\gamma_{\alpha }\\ & \left.\left.+{\tilde{n}}^{2}\gamma_{\alpha }q_{24}N_{i}/\gamma_{\beta }\right]N_{j}+q_{33}\gamma_{\alpha }\gamma_{\beta }N_{i,\zeta }N_{j,\zeta }\right\}d\zeta , \end{array}$$

(A43) $$k_{41}\left(N_{i},\;N_{j}\right)=k_{14}\left(N_{j},\;N_{i}\right),$$

(A44) $$k_{42}\left(N_{i},\;N_{j}\right)=k_{24}\left(N_{j},\;N_{i}\right),$$

(A45) $$k_{43}\left(N_{i},\;N_{j}\right)=k_{34}\left(N_{j},\;N_{i}\right),$$

(A46) $$k_{44}=\int_{-h/2}^{h/2}\left\{-\left[{\tilde{m}}^{2}\right.\right.\gamma_{\beta }\eta_{11}N_{i}/\gamma_{\alpha }\left.+{\tilde{n}}^{2}\gamma_{\alpha }\eta_{22}N_{i}/\gamma_{\beta }\right]N_{j}-\left.\eta_{33}\gamma_{\alpha }\gamma_{\beta }N_{i,\zeta }N_{j,\zeta }\right\}d\zeta ,$$

(A47) $$k_{45}=\int_{-h/2}^{h/2}\left\{-\left[{\tilde{m}}^{2}\right.\right.\gamma_{\beta }d_{11}N_{i}/\gamma_{\alpha }\left.+{\tilde{n}}^{2}\gamma_{\alpha }d_{22}N_{i}/\gamma_{\beta }\right]N_{j}-\left.d_{33}\gamma_{\alpha }\gamma_{\beta }N_{i,\zeta }N_{j,\zeta }\right\}d\zeta ,$$

(A48) $$k_{51}\left(N_{i},\;N_{j}\right)=k_{15}\left(N_{j},\;N_{i}\right),$$

(A49) $$k_{52}\left(N_{i},\;N_{j}\right)=k_{25}\left(N_{j},\;N_{i}\right),$$

(A50) $$k_{53}\left(N_{i},\;N_{j}\right)=k_{35}\left(N_{j},\;N_{i}\right),$$

(A51) $$k_{54}\left(N_{i},\;N_{j}\right)=k_{45}\left(N_{j},\;N_{i}\right),$$

(A52) $$k_{55}=\int_{-h/2}^{h/2}\left\{-\left[{\tilde{m}}^{2}\right.\right.\gamma_{\beta }\beta_{11}N_{i}/\gamma_{\alpha }\left.+{\tilde{n}}^{2}\gamma_{\alpha }\beta_{22}N_{i}/\gamma_{\beta }\right]N_{j}-\left.\beta_{33}\gamma_{\alpha }\gamma_{\beta }N_{i,\zeta }N_{j,\zeta }\right\}d\zeta ;$$

(A53) $$m_{11}=m_{22}=m_{33}=\int_{-h/2}^{h/2}\left\{\rho \gamma_{\alpha }\gamma_{\beta }N_{i}N_{j}\right\}d\zeta ;$$

(A54) $$g_{11}=\int_{-h/2}^{h/2}\left\{c_{\alpha 1}\gamma_{\beta }N_{i}N_{j}/\left(\gamma_{\alpha }R_{\alpha }^{2}\right)\right\}d\zeta ,$$

(A55) $$g_{13}=g_{31}=-\int_{-h/2}^{h/2}\left\{\tilde{m}c_{\alpha 1}\gamma_{\beta }N_{i}N_{j}/\left(\gamma_{\alpha }R_{\alpha }\right)\right\}d\zeta ,$$

(A56) $$g_{22}=\int_{-h/2}^{h/2}\left\{c_{\alpha 2}\gamma_{\alpha }N_{i}N_{j}/\left(\gamma_{\beta }R_{\beta }^{2}\right)\right\}d\zeta ,$$

(A57) $$g_{23}=g_{32}=-\int_{-h/2}^{h/2}\left\{\tilde{n}c_{\alpha 2}\gamma_{\alpha }N_{i}N_{j}/\left(\gamma_{\beta }R_{\beta }\right)\right\}d\zeta ,$$

(A58) $$g_{33}=\int_{-h/2}^{h/2}\left\{c_{\alpha 1}N_{i}N_{j}/\left[\gamma_{\beta }/\left(\gamma_{\alpha }R_{\alpha }^{2}\right)+{\tilde{m}}^{2}\gamma_{\beta }/\gamma_{\alpha }\right]+c_{\alpha 2}N_{i}N_{j}/\left[\gamma_{\alpha }/\left(\gamma_{\beta }R_{\beta }^{2}\right)+{\tilde{n}}^{2}\gamma_{\alpha }/\gamma_{\beta }\right]\right\}d\zeta .$$
